# Genome-wide association mapping of aluminum toxicity tolerance and fine mapping of a candidate gene for *Nrat1* in rice

**DOI:** 10.1371/journal.pone.0198589

**Published:** 2018-06-12

**Authors:** Yonghong Tao, Yanan Niu, Yun Wang, Tianxiao Chen, Shahzad Amir Naveed, Jian Zhang, Jianlong Xu, Zhikang Li

**Affiliations:** 1 Institute of Crop Sciences/National Key Facility for Crop Gene Resources and Genetic Improvement, Chinese Academy of Agricultural Sciences, Beijing, China; 2 Beijing Vegetable Research Center, Beijing Academy of Agricultural and Forestry Sciences, Beijing, China; 3 Shenzhen Institute of Breeding and Innovation, Chinese Academy of Agricultural Sciences, Shenzhen, China; China National Rice Research Institute, CHINA

## Abstract

Aluminum (Al) stress is becoming the major limiting factor in crop production in acidic soils. Rice has been reported as the most Al-tolerant crop and the capacity of Al toxicity tolerance is generally evaluated by comparing root growth under Al stress. Here, we performed an association mapping of Al toxicity tolerance using a core collection of 211 *indica* rice accessions with 700 K high quality SNP data. A total of 21 putative QTL affecting shoot height (SH), root length (RL), shoot fresh weight (SFW), shoot dry weight (SDW), root dry weight (RDW) and shoot water content (SWC) were identified at seedling stage, including three QTL detected only under control condition, eight detected only under Al stress condition, ten simultaneously detected in both control and Al stress conditions, and seven were identified by stress tolerance index of their corresponding traits. Total of 21 candidate genes for 7 important QTL regions associated with Al toxicity tolerance were identified based on combined haplotype analysis and functional annotation, and the most likely candidate gene(s) for each important QTL were also discussed. Also a candidate gene *Nrat1* on chromosome 2 was further fine-mapped using BSA-seq and linkage analysis in the F_2_ population derived from the cross of Al tolerant accession CC105 and super susceptible accession CC180. A new non-synonymous SNP variation was observed at *Nrat1* between CC105 and CC180, which resulted in an amino-acid substitution from Ala (A) in CC105 to Asp (D) in CC180. Haplotype analysis of *Nrat1* using 327 3K RGP accessions indicated that minor allele variations in *aus* and *indica* subpopulations decreased Al toxicity tolerance in rice. The candidate genes identified in this study provide valuable information for improvement of Al toxicity tolerance in rice. Our research indicated that minor alleles are important for QTL mapping and its application in rice breeding when natural gene resources are used.

## Introduction

Aluminum (Al) is the most abundant metal in the Earth’s crust. Under acidic condition (pH<5.0), Al is in the soluble form of trivalent Al^3+^ ion, which is highly toxic to plant growth. Al toxicity is becoming the major limiting factor in crop production, as approximately 30–40% of the world’s arable land is acidic [1]. The root apex is the most sensitive part of the plant to Al and one notable symptom of Al toxicity is the inhibition of root elongation, as the root apex is the site for cell division and expansion [1,2]). Hence, the capacity of Al toxicity tolerance is generally assessed by comparing root growth under Al stress. Several researches have been done on the genetic mechanism of Al toxicity tolerance in rice [3–8], maize [9,10], wheat [11,12], sorghum [13,14] and barley [15,16]. Rice is reported as the most Al-resistant crops under both hydroponic and field conditions. Generally, rice is nearly two to five times more Al toxicity tolerance than other cereals [17]. Due to its relative Al toxicity tolerance, numerous genomic resources and easily growing in hydroponic solution, rice becomes a very good model for investigating the genetics of Al toxicity tolerance.

QTL mapping is a powerful tool in understanding the genetic basis of quantitative phenotypic variation and providing linkage markers in marker-assisted selection (MAS) breeding. Currently, several QTL has been identified [3,4,6,18,19], and four mutant genes that lead to Al sensitivity have been cloned in rice, such as *Nrat1*, *STAR2*, *ART1* and *STAR1* [20]. However, conventional QTL analysis has been time-consuming and labor-intensive mainly because it requires development of polymorphic markers and mapping population. To overcome these limitations, genome wide association study (GWAS) was introduced as a new approach in gene identification and QTL mapping in plants, which widely used for natural resources. Depending on large amount of SNP markers, GWAS was faster and more accurate in determination of recombination breakpoints.

Bulked segregant analysis (BSA) is another simple and rapid method to identify molecular markers tightly linked to the causal gene for a given trait [21]. It was based on the co-segregation between the markers and targeted genes in two groups of individuals with extreme phenotypes. This method was appropriate to the qualitative traits controlled by one gene or quantitative traits controlled by major QTL. With the development of high-throughput sequencing in recent years, whole genome sequencing with high-efficient and low-cost is available. Moreover, the combination of whole genome sequencing with BSA method was named as BSA-seq by Takagi et al. [22], which can accurately identify the location of QTL and major genes associated with objective trait by large amount of single nucleotide polymorphism (SNP) after whole genome sequencing of bulked samples. Nowadays, BSA-seq as a rapid approach for identifying QTL has been used in rice [22,23], cotton [24], cucumber [25–27] and maize [28].

Although rice is known as the most tolerant species to Al toxicity among cereal crops, there is widely genotypic variation in its tolerance to Al stress [29]. High Al concentration and long time of Al stress are needed to observe significant differences between Al resistant and susceptible varieties. To the best of our knowledge, QTL analysis of Al toxicity tolerance associated with up ground traits at seedling stage are poorly understand in rice. In the present study, we evaluated Al toxicity tolerance using its related traits in both natural germplasm resources and a F_2_ population derived from the cross of two extreme accessions, to elucidate the genetic basis and identify the candidate genes of Al toxicity tolerance by both GWAS and BSA-seq analysis methods.

## Materials and methods

### Plant materials

A total 222 *indica* rice accessions which were a subset introduced from a core collection established at International Rice Research Institute (IRRI). As our previous research [30], the genetic distance of 11 accessions were different from others by principal component analysis (PCA) analysis. Hereinto, 211 accessions were used to evaluate the Al toxicity tolerance under high concentration of Al for a long time. CC105 (PR) and CC180 (PS), of which the first was identified as Al tolerant accession with the tolerant allele at the major QTL in the range of 4.61–4.86 Mb on chromosome 2 based on GWAS analysis while the latter as Al super susceptible accession, were used to develop F_2_ population for BSA-seq and linkage analyses. Moreover, 327 rice accessions consisted of many ecotypes were selected from 3K RGP [31] and used for haplotype analysis of candidate gene *Nrat1*.

### Evaluation for Al toxicity tolerance and phenotypic data collection

All experiments in this study were conducted in a greenhouse at of Institute of Crop Sciences of CAAS in Beijing. Evaluation for Al toxicity tolerance in 211 *indica* rice accessions were performed under both control and Al stress conditions in May 2015. The first F_2_ population (300 seedlings) only under Al stress condition was conducted for Al evaluation in October 2016. The second F_2_ population (500 seedlings) only under Al stress and 327 3K accessions under both control and Al stress conditions for Al stress evaluations were performed in May 2017. A randomized complete block design was used with two replications for each accession. Screening for Al toxicity tolerance of all materials was conducted according to the following steps. Rice seeds were soaked in distilled water in the dark at 30°C for 48 hours after sterilized with 5% sodium hypochlorite solution for 20 minutes. Then, seeds with uniform germination were directly sown in holes of perforated styrofoam sheets (10 lines × 13 rows) with a nylon net bottom in the plastic container (45 cm × 32 cm × 17 cm) with water. Five days later, all seedlings were exposed to Yoshida solution [32] containing 0 mM L^-1^ Al (AlCl_3_·6H_2_O) for control condition and 1 mM L^-1^ Al (AlCl_3_·6H_2_O) for stress condition. The pH of the solution was adjusted to 4.5 at alternative day by 1 M NaOH / HCl and solution was renewed every five days. The day/night temperature in green house was around 32/24°C and the relative humidity was ~50%. The growth condition was regulated by using shade net and mechanical ventilation along with the cooling pad. After 3 weeks of Al treatment, all seedlings were harvest and shoot height (SH) and root length (RL) of each plant was measured with a ruler. For 211 *indica* rice accessions, we also weighed shoot fresh weight (SFW), and then the samples were kept in oven under 50°C for 72 h. Finally, root dry weight (RDW, mg) and shoot dry weight (SDW, mg) were recorded, and shoot water content (SWC, %) was calculated by (SFW– SDW) / SFW × 100. For each trait, Al stress tolerance index was determined by trait ratio of stress to control conditions. Except for the two F_2_ individual populations, the extreme values for each trait which data falled outside the range of (mean of all accessions ± 3 times the standard deviation) were removed and the trait value was taken from the average of two replicates. Trait pair-wise correlation and analysis of variance was performed by SAS8.1 PROC CORR and GLM, respectively.

### Association mapping of Al toxicity tolerance

We carried out GWAS to detect SNP-trait associations for all measured traits using 395, 553 SNP markers and 211 accessions. The genotyping and distribution of SNP markers used in this study were described in our previous research [30]. A mixed linear model (MLM) was applied in GAPIT [33], a package of R software incorporating kinship matrix and seven PCA axes, which would be enough to explain 90% of the variance in the natural population. Significant marker trait associations were determined based on a threshold of *p*-value 1.0×10^−4^. This threshold was justified by Quantile-Quantile plot (S1 File) and was also applied by our previous researches using the same population [30]. Adjacent significant SNP associated with same trait within a physical distance of 200 kb were regarded as a single QTL.

### Candidate genes analysis for important QTL affecting Al toxicity tolerance

QTL meeting at least the following two items was considered to be important: (1) associated with different traits; (2) identified under both normal and Al stress conditions with same direction of gene effect and similar magnitude; (3) affecting the relative trait ratio of stress to control conditions; (4) neighbor to previously reported QTL. Then candidate genes analysis for important QTL was carried out by the following four steps. Firstly, for each important QTL region, the SNPs whose –log_10_(*p*) located inside the interval of 1 unit of the peak SNP were regarded as significant. Secondly, all significant SNPs were used to check non-synonymous mutation in the coding sequence (CDS) region for all the genes located in the interval of each important QTL from the Rice Genome Annotation Project (RGAP). Thirdly, if more than two significant SNPs distributed in one gene, haplotype analysis was carried out for each of the candidate genes in each important QTL region using all non-synonymous SNPs located inside of the gene CDS region. Finally, candidate genes were determined by testing the significant differences among major haplotypes (containing more than 10 samples) for each important QTL through ANOVA.

### BSA-seq analysis of the major QTL on chromosome 2

To validate the major QTL for Al toxicity tolerance identified on chromosome 2, we developed a F_2_ population by crossing of PR with PS. Two DNA bulks, Al-resistant bulk (BR) and Al-susceptible bulk (BS) using respective 20 individuals selected from the 330 individuals in the F_2_ population by mixing an equal amount of genomic DNA. The DNA of PR, PS, BR and BS were randomly sheared by ultrasonic and the resulting fragments were ligated with adapters. Each DNA library was sequenced using an Illumina Hi-seq 2000 sequencing platform. Low-quality paired reads were removed from the raw reads according to the following characters: (1) the reads with adapter; (2) single read has more than 10% of missing bases; (3) single read has more than 50% of bases with alignment Q-score lower than 5. The clean reads selected by the quality criteria were then mapped to the *japonica* cv. Nipponabre reference genome (RGAP 7, http://rice.plantbiology.msu.edu/) by BWA software. SNP-calling and SNP-annotation were performed by SAM tools and ANNOVAR software, respectively [34,35].

For BSA-seq analysis, SNP-index of DNA bulks were obtained by using high-quality polymorphic SNPs between PR and PS. SNP-index was the ratio of short reads harboring SNPs different from reference genome [36], and Δ(SNP-index) was the difference of SNP-index between BR and BS. Furthermore, to reduce the effect of sequencing or alignment errors, some SNP sites were filtered with the following criteria: (1) the SNP-index in two bulks < 0.3 and read coverage depth < 7X, and (2) the SNP-index was missing in one bulk. The distribution of SNP-index in the whole genome was calculated using sliding window analysis method (window size 1 Mb and step size 1 kb). We performed 1000 permutations for Δ(SNP-index) on the whole genome and selected 95% confidence intervals to identify candidate regions for Al-tolerance QTL [22].

### Linkage analysis of the major QTL on chromosome 2

The major gene for Al toxicity tolerance in the region of 0.16–2.79 Mb on chromosome 2 identified by BSA-seq analysis was fine-mapped using selective genotyping method. Based on the polymorphic SNP between PR and PS, we further designed 11 KASP markers in the region of 0.16–2.79 Mb on chromosome 2 and applied to 186 extreme individuals (93 extreme tolerant individuals and 93 susceptible ones) selected from 1000 F_2_ individuals for fine mapping of the major gene. Linkage mapping analysis was carried out by IciMapping V4.1 software [37]. Detailed information of all KASP markers used for linkage analysis was shown in S2 File.

### Sequence and expression analysis of *Nrat1* in PR and PS

A previously reported gene *Nrat1* involved in Al transportation was detected by BSA-seq in this study. To examine the expression level of *Nrat1* in PR and PS, total RNA was extracted using an RNeasy Mini Kit (Qiagen) from the roots of 5-d-old seedlings exposed to Al stress for 0, 2, 12 and 24 h. The first strand cDNA synthesis and RT-PCR examination were completed by using SYBR Green PCR Master Mix kit (Applied Biosystems, USA) following the manufacturer’s instructions. The full-length cDNA sequence of *Nrat1* in PR and PS was amplified by PCR using the gene specific primers. The 2 kb region upstream of the start codon of *Nrat1* was recognized as promoter region and amplified by three paired primers from both PR and PS genomic DNA. Primer sequences of *Nrat1* for RT-PCR were designed from a website Quant Prime (http://quantprime.mpimp-golm.mpg.de/). The relative transcript levels of *Nrat1* and *Ubi* (internal control) were determined by quantitative real-time RT-PCR, which was calculated using the 2^-ΔΔCt^ method [38]. The differences in expression levels among PR and PS were analyzed by t-test. Each test was repeated for two times biologically and three times technically. The primer sequences mentioned above are also shown in S2 File.

### Haplotype analysis of *Nrat1* in 327 accessions from 3K RGP

All SNPs in the promoter region (2 kb) of *Nrat1* and non-synonymous SNPs located inside of *Nrat1* were selected from 32 M SNPs data from the Rice SNP-Seek Database [39]. The SNPs with missing rate over 20% and MAF less than 5% were filtered and the remaining SNPs were used for haplotype analysis of *Nrat1*.

## Results

### Phenotypic variations responsive to Al stress

**Fig 1:**
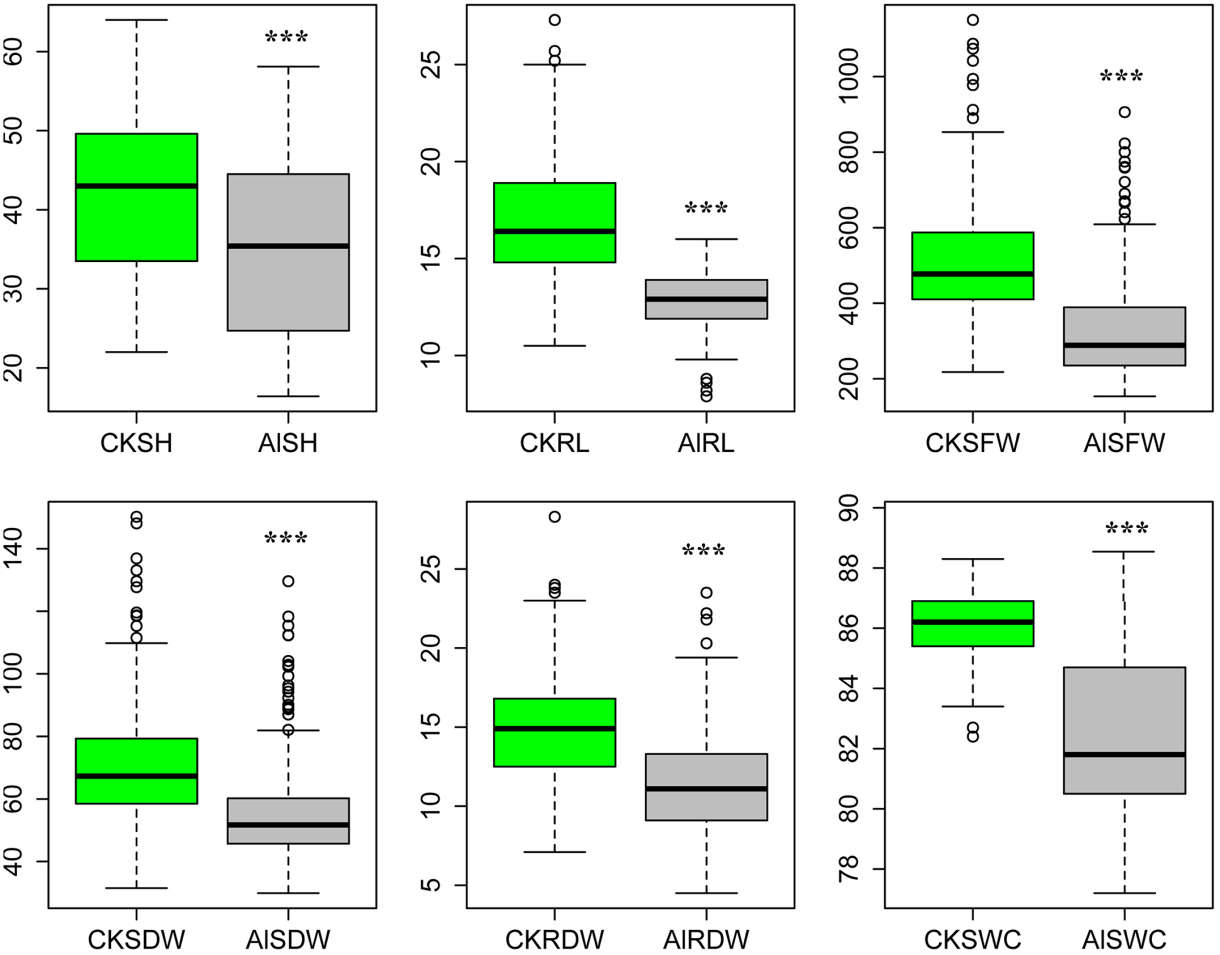
Box plot of six measured traits for 211 *indica* accessions under normal and Al stress conditions. CK, control condition; Al, Al stress condition; SH, shoot height; RL, root length; SFW, shoot fresh weight; SDW, shoot dry weight; RDW, root dry weight; SWC, shoot water content. *** indicated significance of ANOVA at p < 0.001.

In the current association panel, 211 *indica* rice accessions were evaluated for Al toxicity tolerance with 1 mM Al^3+^ in hydroponic nutrient solution. In comparison with control condition, the mean value of all six measured traits was significantly decreased under Al stress condition (Fig 1). ANOVA analysis indicated that significant differences were observed among the environments and genotypes for all investigated traits. Environment accounted for an average of 87.7% of phenotypic variance ranging from 75.6% in RL to 96.6% in SH (S1 Table), while genotype explained an average of 90.7% of phenotypic variance ranging from 72.9% of Al/CKRL to 99.9% of CKSWC (S2 Table). High and positive correlations were observed among SH, SFW and SDW under both control and Al stress conditions, suggesting that seedling had similar mechanism of biomass accumulation in different environments. Except for RL and SWC, significant positive correlations were found for the same traits under control and Al stress conditions, indicating that Al toxicity mainly affected the root elongation and absorbing water at seedling stage (S1 Fig).

**Fig 2:**
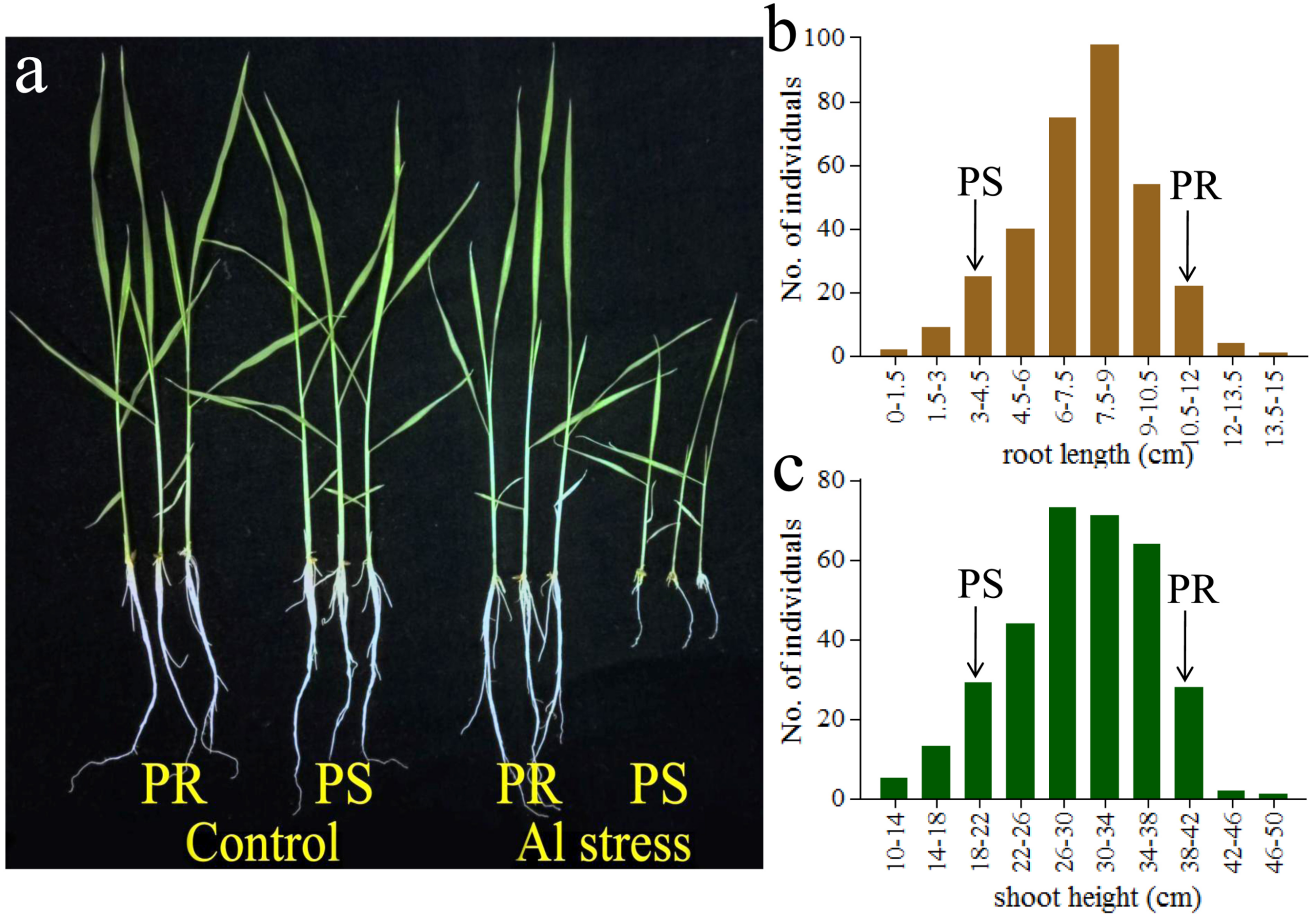
Performance of the two parents and F_2_ population after treatment of Al stress. (a) Phenotypic difference of two parents under control and Al stress conditions. (b-c) Frequency distribution of root length (RL) and shoot height (SH) in the F_2_ population derived from the two parents under Al stress condition.

As compared with PR, PS exhibited an extremely sensitive to Al stress (Fig 2a). The SH (RL) ratio of Al stress to control conditions was 0.92 (0.97) in PR and 0.61 (0.34) in PS, respectively, indicating that RL and SH of PS were obviously decreased under Al stress, while no significant differences were found of PR between control and stress conditions. The frequency distribution of RL and SH under Al stress conditions among 330 F_2_ individuals were presented in Fig 2b and 2c. The SH and RL in F_2_ population showed continuous variations, suggesting that Al stress tolerance in rice is quantitatively inherited.

### Identification of Al toxicity tolerance QTL through GWAS

**Fig 3:**
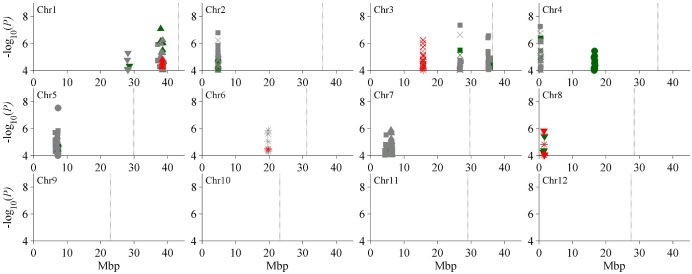
Manhattan plots of QTL for aluminum toxicity tolerance in the whole genome. Significant SNPs from different conditions are displayed in different colors: control is green, aluminum stress is grey, the ratio of stress to control is red. The associated traits are represented by different symbols: shoot height = triangle up, root length = triangle down, shoot fresh weight = ×, shoot dry weight = square, root dry weight = circle, shoot water content = star.

In 211 *indica* rice accessions, a total of 21 QTL were identified for SH, RL, SFW, SDW, RDW and SWC, including three detected only under control condition, eight detected only under Al stress condition, and ten simultaneously detected in both control and Al stress conditions. Among these, seven QTL (*qSh1*, *qRl8*, *qSfw3a*, *qSdw3a*, *qSwc3*, *qSwc6* and *qSwc8*) were identified by stress tolerance index of their corresponding traits (Table 1, Fig 3).

**Table 1:** QTL identified with significant association to Al toxicity tolerance related traits.

Trait^a^	QTL	Chr	Position (Mb)	Control			Al stress			Al stress/CK ratio			Previously reported QTL or gene
				P value ^b^	Effect ^c^	R^2^ (%) ^d^	P value	Effect	R^2^ (%)	P value	Effect	R^2^ (%)	
SH	*qSh1*	1	37.93–38.73	8.07E-08	-4.5	6.2	5.76E-07	-4.9	5.7	1.7E-05	-0.06	7.8	*OsMGT1*[43]
	*qSh7*	7	6.15–6.38	2.66E-05	3.1	3.7	1.28E-06	3.2	5.3				*QAlRr7*.*1*[19]
RL	*qRl1*	1	28.15–28.16				5.02E-06	-1.1	9.6				
	*qRl8*	8	1.28–1.67	3.81E-06	2.3	9.0				1.43E-06	-0.14	11.0	
SFW	*qSfw2*	2	4.74–4.86				5.93E-07	-53.2	7.6				
	*qSfw3a*	3	15.59–15.77				1.65E-05	81.6	5.5	5.51E-07	0.10	10.8	*PSM377*[7]
	*qSfw3b*	3	26.62–26.79	7.39E-06	101.4	7.0	2.26E-07	92.1	8.2				
	*qSfw4*	4	0.38–0.61	3.40E-06	96.5	7.6	8.82E-08	88.9	8.7				
SDW	*qSdw1*	1	37.02–37.60				1.17E-06	13.8	8.0				
	*qSdw2*	2	4.61–4.86	2.46E-05	-7.3	6.7	1.61E-07	-7.3	9.4				
	*qSdw3a*	3	26.52–27.07	3.16E-06	13.6	8.3	4.49E-08	12.7	10.3	7.09E-05	-0.04	7.7	
	*qSdw3b*	3	35.01–35.79	2.61E-05	11.2	6.7	2.78E-07	12.3	9.0				
	*qSdw4*	4	0.38–0.61	4.65E-07	13.7	9.8	5.44E-08	11.8	10.2				
	*qSdw5*	5	6.46–7.54	3.36E-05	10.7	6.5	1.95E-06	11.3	7.7				*qAlSDW5*[8]
	*qSdw7*	7	4.30–4.75				2.96E-06	-11.9	7.4				
RDW	*qRdw2*	2	4.74–4.76	2.12E-05	-1.2	8.4							*QAlSr2*.*1*[8]
	*qRdw4*	4	16.39–16.67	3.61E-06	2.0	10.0							
	*qRdw5*	5	7.01–7.32	1.90E-05	2.2	8.5	2.89E-08	2.9	15.3				
SWC	*qSwc3*	3	15.62–15.74				2.77E-05	1.1	6.1	1.48E-05	0.01	7.6	*PSM377*[7]
	*qSwc6*	6	19.60–19.76				2.52E-06	1.6	7.8	3.09E-05	0.02	7.0	
	*qSwc8*	8	1.30–1.58				1.44E-05	2.5	6.6	2.16E-06	0.03	9.2	*qALRR-8*[45]

^a^ Same as in Fig 1.

^b^ The peak value in the chromosome region.

^c^ Allele effect with respect to the minor allele.

^d^ Phenotypic variance explained.

Two QTL (*qSh1* and *qSh7*) affecting SH were simultaneously identified on chromosomes 1 under both control and stress conditions, explaining phenotypic variance of 6.2% (5.7%) and 3.7% (5.3%) under control (stress) conditions, respectively. And *qSh1* also affected stress tolerance index of SH with 7.8% of phenotypic variance (Table 1).

For RL, two QTL (*qRl1* and *qRl8*) were identified on chromosomes 1 and 8. *qRl1* was only identified under stress condition and account for 9.6% of phenotypic variance while *qRl8* was detected under control condition with 9.0% of phenotypic variance, and it simultaneously affected stress tolerance index of RL with 11.0% of phenotypic variance (Table 1).

Four QTL governing SFW were identified on chromosomes 2, 3 and 4. *qSfw2* and *qSfw3a* were detected under stress condition, explaining 7.6% and 5.5% of phenotypic variance, respectively. Moreover, *qSfw3a* also affected stress tolerance index of SFW with 10.8% of phenotypic variance. *qSfw3b* and *qSfw4* were detected under both stress and control conditions and account for 7.0% (8.2%) and 7.6% (8.7%) of phenotypic variance in control (stress) conditions, respectively (Table 1).

Seven QTL governing SDW were identified on chromosomes 1, 2, 3, 4, 5 and 7. *qSdw1* and *qSdw7* were detected only under stress condition, explaining 8.0% and 7.4% of phenotypic variance, respectively. The other five QTL (*qSdw2*, *qSdw3a*, *qSdw3b*, *qSdw4* and *qSdw5*) were identified under both control and stress conditions and accounted for phenotypic variance ranging from 6.5% to 10.3%. What’s more, *qSdw3a* also affected stress tolerance index of SDW with 7.7% of phenotypic variance (Table 1).

For RDW, 3 QTL were detected on chromosomes 2, 4 and 5. *qRdw2* and *qRdw4* were detected only under control condition and explained 8.4% and 10.0% of phenotypic variance, respectively. *qRdw5* was identified under both control and stress conditions and explained 8.5% and 15.3% of phenotypic variance, respectively (Table 1).

Three QTL (*qSwc3*, *qSwc6* and *qSwc8*) affecting SWC were identified under stress condition, accounting for 6.1%, 7.8% and 6.6% of phenotypic variance, respectively. These all three QTL had significant effects on Al stress tolerance index of SWC and explained 7.6%, 7.0% and 9.2% of phenotypic variance, respectively (Table 1).

### Candidate genes analysis for the important QTL

**Fig 4:**
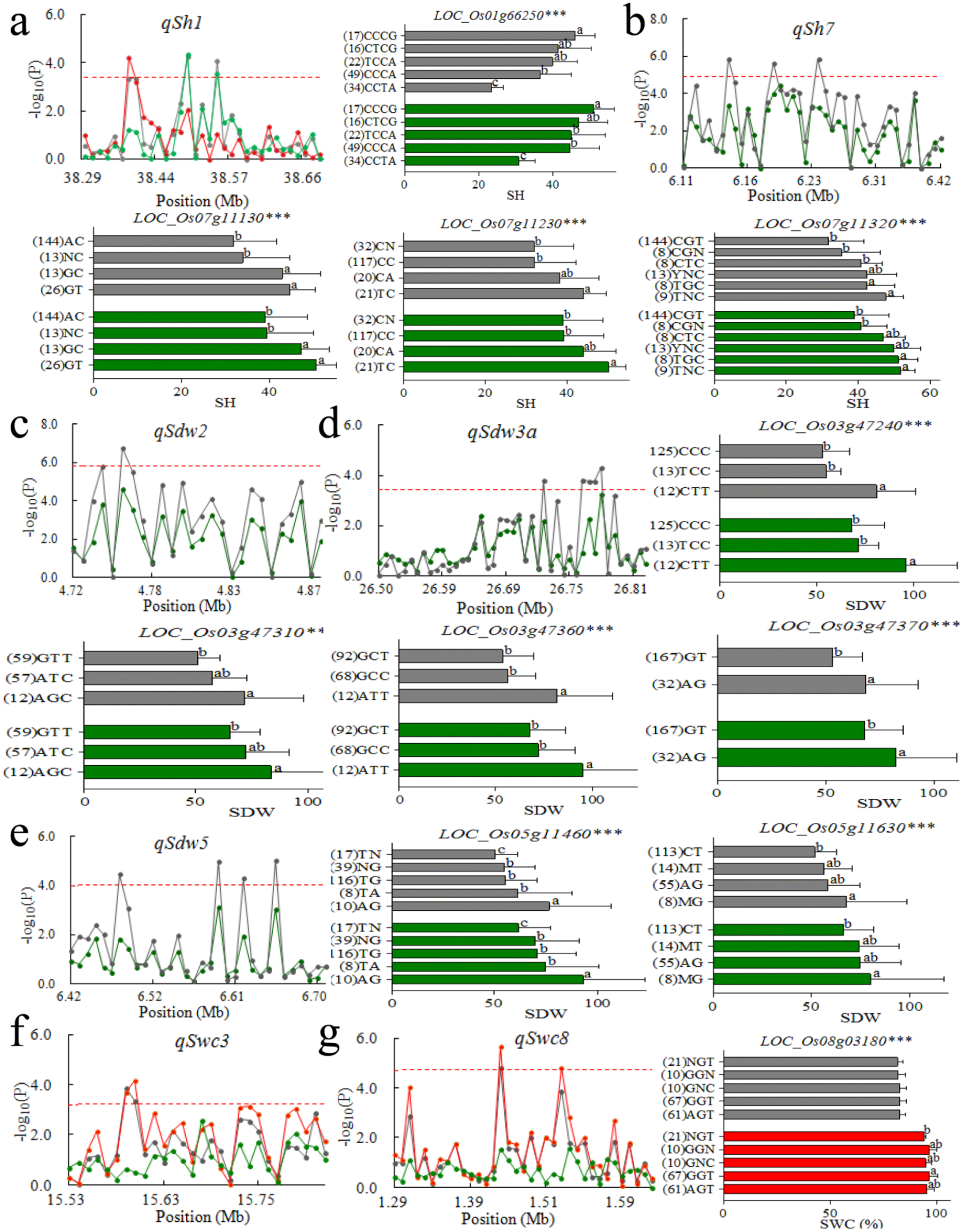
Manhattan plot of important QTL and haplotype analysis of candidate genes related to QTL including *qSh1* (a), *qSh7* (b), *qSdw2* (c), *qSdw3a* (d), *qSdw5* (e), *qSwc3* (f) and *qSwc8* (g). Each point was a gene in the region of the QTL. Line and histogram in different colors indicated different conditions: green is control condition, grey is Al stress condition and red is the ratio of the stress to control conditions. Dash line showed the threshold to determine candidate genes. The ** and *** suggested significance of ANOVA at p < 0.01and p < 0.001, respectively. The letter on histogram (a and b) indicated multiple comparisons result at the significant level 0.01. The value in brackets was the number of individuals for each haplotype.

A total of 21 candidate genes in 7 important QTL regions were identified, ranging from 1 to 5 candidate genes for each important QTL. Only 11 candidate genes had non-synonymous SNPs located in the CDS region and were conducted for haplotype analysis (Fig 4).

For *qSh1* in the region 38.29–38.66 Mb on chromosome 1, 145 SNPs were identified in 33 genes, and 3 candidate genes (*LOC_Os01g66100*, *LOC_Os01g66250* and *LOC_Os01g66360*) were associated with significant SNPs for *qSh1*. Significant differences for SH were found between the five haplotypes in candidate genes *LOC_Os01g66250*, and the haplotype CCTA significantly decrease SH than the other four haplotypes. No haplotype was found for the other two genes resulting from non-synonymous SNPs detected in the CDS regions of them (Fig 4a).

For *qSh7* in the region from 6.11 to 6.42 Mb on chromosome 7, 150 SNPs were identified in 41 genes. Three candidate genes (*LOC_Os07g11130*, *LOC_Os07g11230* and *LOC_Os07g11320*) were associated with significant SNPs for *qSh7*. Haplotype analysis revealed that minor alleles in all these three genes significantly increased SH under both control and stress conditions, which had same effect as the peak SNP (Fig 4b).

For *qSdw2* located in the region 4.72–4.87 Mb on chromosome 2, 109 SNPs were detected in 27 genes. *LOC_Os02g09220* and *LOC_Os02g09240* were the only two candidate genes for *qSdw2*. No haplotype was found for these two genes (Fig 4c).

For *qSdw3a* in the region from 26.5 to 26.81 Mb on chromosome 3, 155 SNPs were identified in 43 genes, and 5 candidate genes (*LOC_Os03g47240*, *LOC_Os03g47310*, *LOC_Os03g47330*, *LOC_Os03g47360* and *LOC_Os03g47370*) were found. Among them, no haplotype was found in *LOC_Os03g47330* (Fig 4d). Significant differences for SDW were found between the haplotypes in the four candidate genes *LOC_Os03g47240*, *LOC_Os03g47310*, *LOC_Os03g47360* and *LOC_Os03g47370* under both control and stress conditions.

For *qSdw5* in the region 6.42–6.70 Mb on chromosome 5, 124 SNPs were found in 32 genes. Four candidate genes (*LOC_Os05g11460*, *LOC_Os05g11630*, *LOC_Os05g11660* and *LOC_Os05g11730*) were observed for *qSdw5*, but no haplotype was found for *LOC_Os05g11660* and *LOC_Os05g11730*. The minor haplotype significantly increased SDW in *LOC_Os05g11460* and *LOC_Os05g11630* (Fig 4e).

For *qSwc3* in the region of 15.62–15.74 Mb on chromosome 3, 93 SNPs were identified in 28 genes. *LOC_Os03g27230* and *LOC_Os03g27250* were the only two candidate genes for *qSwc3*. However, no haplotype was found for these two genes (Fig 4f).

For *qSwc8* in the region from 1.30 to 1.58 Mb, 168 SNPs were detected in 35 genes. Two candidate genes *LOC_Os08g03180* and *LOC_Os08g03290* were observed for *qSwc8*. Four haplotypes were identified for *LOC_Os08g03180* with significant differences in SWC. No haplotype was found for *LOC_Os08g03290* (Fig 4g).

### Detection of QTL for Al toxicity tolerance by BSA-seq and linkage analysis

For BSA-seq, two parents and two DNA pools were sequenced through Illumina high-throughput sequencing. After trimming and filtering, over 99.9% of raw reads were selected with an average of 95.8% Q20 reads. We ultimately obtained 5.15 G (~13X coverage depths) and 9.15 G (~22 X coverage depths) clean reads with 150 bp in length for two parents and two DNA pools, respectively (S3 Table). About 96.4% of these reads were mapped to the reference genome and 575, 977 SNPs were identified from the two parents (S3 Table).

**Fig 5:**
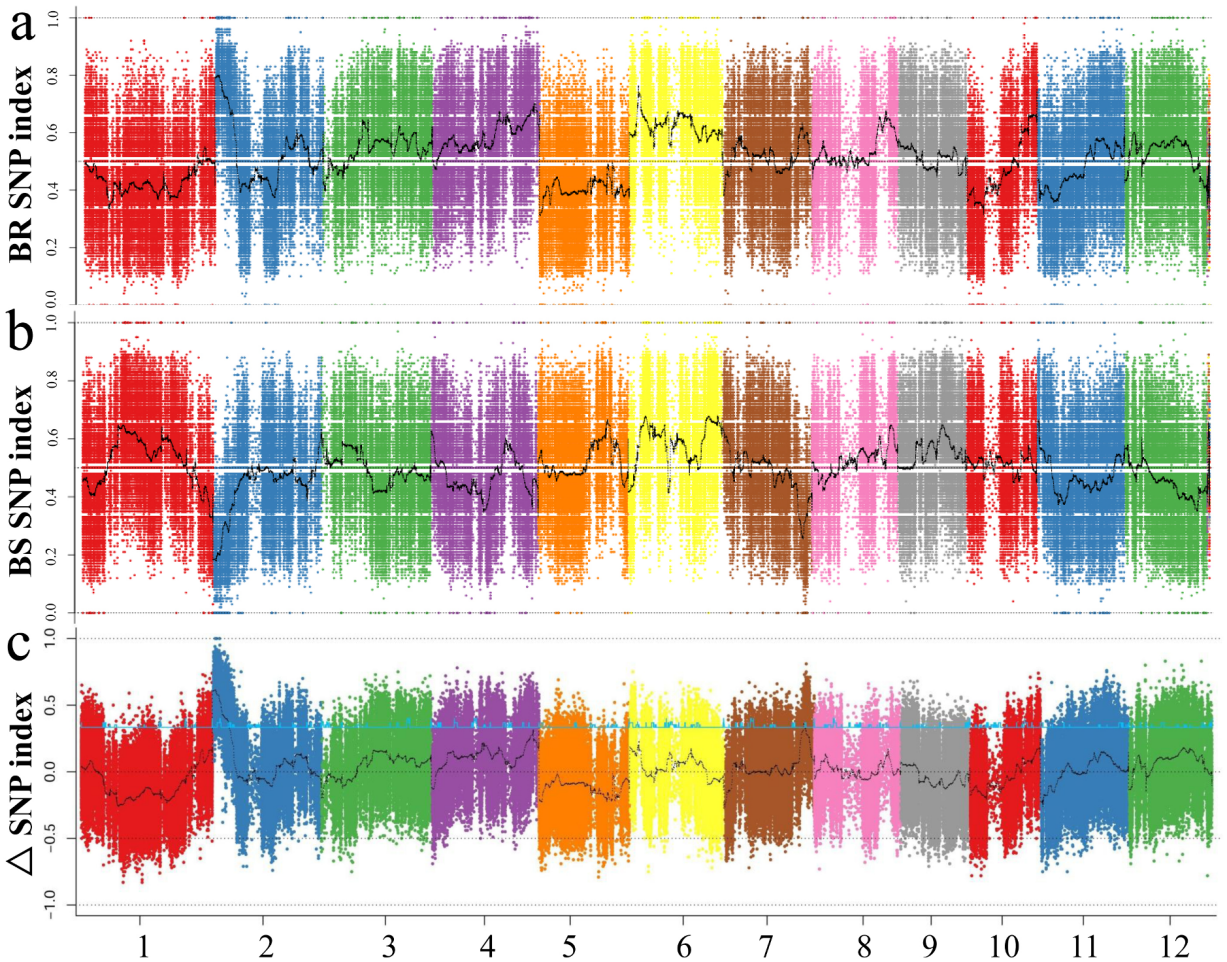
Variation tendency of SNP index (a-b) and ΔSNP index (c) between BR and BS along the genome.

Based on the chosen requirements for SNP, a total of 568, 239 (98.7%) polymorphic SNPs were used to calculate SNP index. We also compared these polymorphic SNPs with 700 K high density SNP data [40], 36, 864 SNP in total is overlapping, and about 94.6% and 98.8% SNPs have same alleles in PR and PS, respectively, indicating the high accuracy of sequencing in this study. However, the density (the number of SNP in 1 Mb) of these SNP varied greatly across the whole genome owing to that these were selected from two *indica* varieties. For BSA-seq, an average SNP-index was calculated for BR and BS in 1 Mb interval with a 1kb sliding window. ΔSNP-index was also computed by subtraction of SNP-index of BR from that of BS. SNP-index in BR, BS and ΔSNP-index were plotted on the whole genome (Fig 5). As described by Abe et al., the SNP-index would equal to 1 near the causal gene and 0.5 for unlinked loci [36]. Thus, the QTL for Al toxicity tolerance on the genomic region would exhibit opposite variation of SNP-index between BR and BS. Based on the trend of ΔSNP index on the whole genome, we defined 0.5 as a threshold to identify candidate region for Al toxicity tolerance. A region of 0.16–2.79 Mb on chromosome 2 was detected with the average SNP-index of BR was 0.79 while the average SNP-index of BS was 0.19 suggesting that there may be a major QTL for Al toxicity tolerance (Fig 5). In this region, a total of 6 SNP sites with ΔSNP-index equal to 1 which SNP index value in BR is 1 and that in BS is 0. The position of these 6 SNPs and near genes was listed in S3 Table. Through analyzing the position of these 6 SNPs, 4 were in the upstream (1kb) of one gene, 2 were in the intergenic region of two genes (S3 Table). In addition, a reported gene *Nrat1* (Nramp aluminium transporter 1) [41], which belongs to the Nramp family and specific for trivalent Al ion transportation was exactly located in this candidate region.

**Fig 6:**
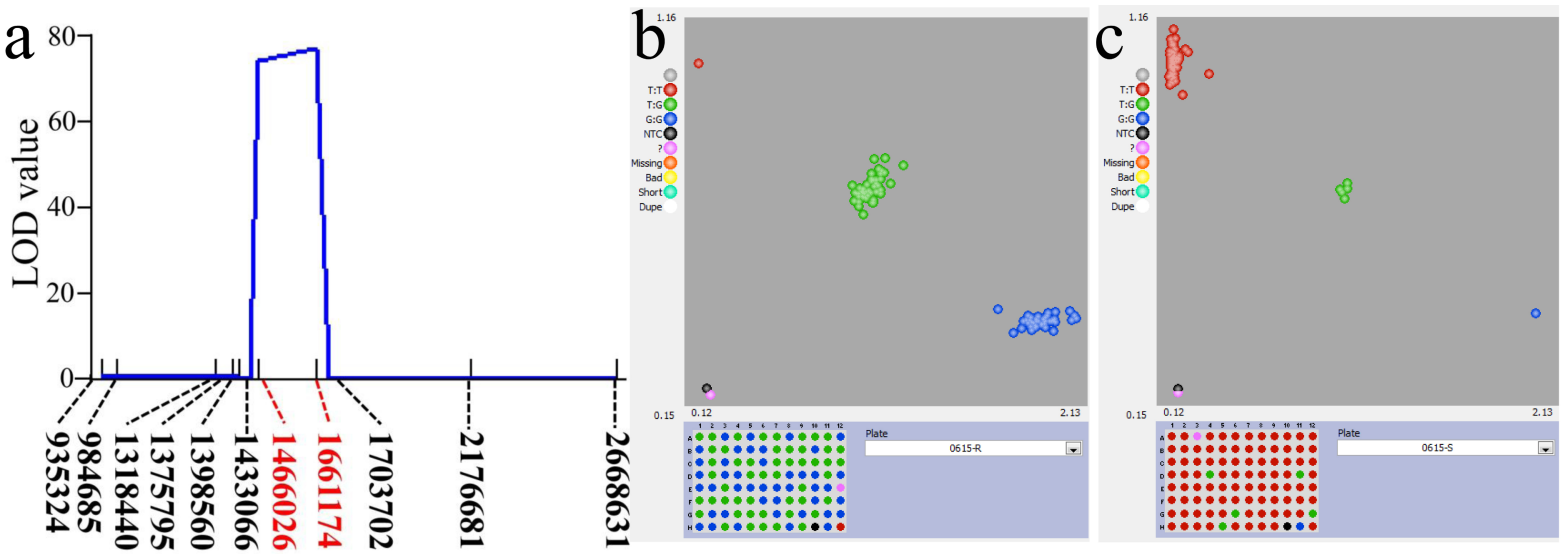
Validation of the major QTL for Al toxicity tolerance by linkage analysis with 11 KASP SNP markers (a), genotype of 93 extremely Al toxicity tolerance (b) and sensitive (c) individuals by SNP1661173, the last three sites were NTC, PR and PS.

To examine whether *Nrat1* is responsible for this major QTL identified by BSA-seq, we additionally evaluated 1000 F_2_ seedlings under Al stress condition in May 2017. To minimize phenotypic errors of each plants, we selected only 186 extremely individuals for a linkage mapping analysis using 11 KASP SNP markers which were designed from the polymorphic SNPs in the region of 0.16–2.79 Mb on chromosome 2 between the two parents. QTL analysis detected a very high LOD (77.03) curves between SNP1466026 and SNP1661174 in the candidate region (Fig 6a). The peak SNP location was completely consistent with *Nrat1* which was involved in Al toxicity tolerance in rice. In fact, there was only one SNP variation in *Nrat1* between PR and PS which was located at 1661173 bp by analyzing the promoter and gene region from ~13× sequencing data. To verify that SNP1661173 was the causal SNP for Al sensitive phenotype, we examined the genotyping result of extreme seedlings by this SNP. There was no homozygous PR allele in Al susceptible bulk and no homozygous PS allele in Al resistant bulk. Total of 42 (46.7%) Al tolerant individuals had homozygous PR allele (Fig 6b) and 87 (96.7%) Al sensitive individuals carried homozygous PS allele (Fig 6c). These results indicated that PS carries a recessive gene underlying Al susceptibility in rice.

### Sequence and expression of *Nrat1* in PR and PS

BSA-seq and linkage mapping inferred that *Nrat1* was the candidate gene for Al toxicity tolerance. The gene, *Nrat1*, was 4069 bp in length and consisted of 13 exons, with 554 amino acid residues. We amplified the full length cDNA and promoter of *Nrat1* from two parents by specific primers. The sequence difference between PR and PS in *Nrat1* by PCR was the same to Illumina sequencing results, which only one SNP variation was found in CDS region. This single-nucleotide transition (G in PR and T in PS) was located in the fourth exon at the position 311 bp from the ATG start codon, resulting in the 104^th^ amino-acid residue changing from Ala (GCC) to Asp (GAC). Compared with the previous SNP variations of *Nrat1* in rice [20,29,42], complete different SNP position was detected in our research, indicating that the SNP identified in this study was a new non-synonymous mutant in *Nrat1* and may play an important role in susceptibility to Al stress as a new allele.

We examined the dynamic expression of *Nrat1* by RT-PCR in both PR and PS after Al treatments at 0, 2, 12 and 24 h. Two hours after treatment with 1 mM L^-1^ Al of seedlings, there was no difference in the expression level of *Nrat1* in root between PR and PS. However, the expression level of *Nrat1* in PR was significantly higher than PS at both 12 h and 24 h, suggesting that higher expression of *Nrat1* was responsible for Al tolerance (S2 Fig).

### Allele variation and haplotype analysis of *Nrat1*

**Fig 7:**
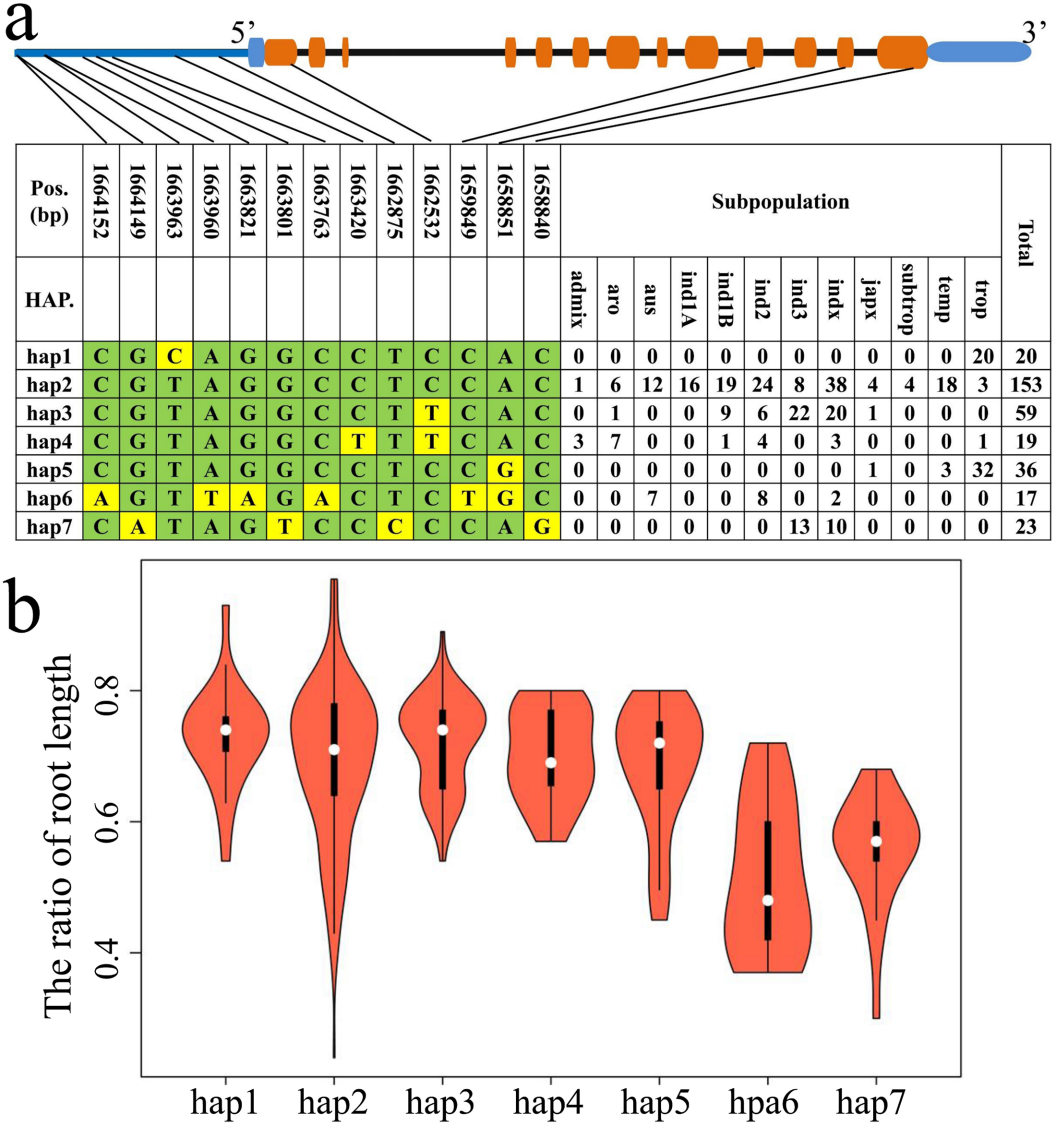
Haplotype analysis in the region of *Nrat1*. (a) Haplotypes of *Nrat1* observed in 327 accessions using 32M SNP data. (b) The performance of root ratio between Al stress to control condition in seven haplotypes.

In this study, we detected a significant signal of *Nrat1* by BSA-seq but cannot be identified by GWAS. To investigate the causative SNP variation in *Nrat1* responsible for the phenotypic variation for tolerance, we used all available SNPs around the *Nrat1* from 32 M SNPs data in the Rice SNP-Seek Database [31]. A total of 174 SNPs in the CDS region and 54 SNPs in the promoter region (2 kb) were observed in *Nrat1*. However, only 4 non-synonymous SNP and 9 SNP in promoter region were selected after filtering the minor allele frequency (MAF) <0.05. Using these 13 SNP data, seven distinct haplotypes were found across the 327 diversity rice accessions. The SNP variations in promoter region were in accordance with the CDS region, indicating that the *Nrat1* promoter and coding regions both play important role in regulating Al toxicity tolerance in rice. Haplotypes 6 and 7 had more allele variations in *Nrat1* and were unique to the *aus* and *indica* subpopulations (Fig 7a and 7b). Through ANOVA analysis, haplotypes 6 and 7 significantly decreased the ratio of RL, indicating that *aus* and *indica* had lower level of Al toxicity tolerance than the other subpopulations in rice [20].

## Discussion

### Identification of QTL and candidate gene for Al toxicity tolerance by GWAS

In the present study, 21 QTL affecting Al toxicity tolerance was identified by associated mapping. Seven of them were found to adjacent to the pervious reported QTL or cloned genes related to Al stress tolerance. For instance, *qSh1* affecting SH detected in the region 37.93–38.73 Mb co-localized with *qAlSL1*.*2* for shoot length, which was identified by MAGIC populations under Al stress treatment [8]. This QTL was also 300 kb away from a known gene *OsMGT1*, which is a functional transporter of Mg and its up-regulation leads to increase Al toxicity tolerance in rice [43]. *qSdw2* and *qSdw7* associated with SDW were adjacent to the two previously mapped QTL, *QAlSr2*.*1* and *QAlRr7*.*1* for Al toxicity tolerance related trait, root growth, respectively [19]. *qSdw5* for SDW located in the region 6.46–7.54 Mb on chromosome 5 was 1Mb away from a reported QTL *qAlSDW5* for shoot dry weight [8]. *qSwc3* and *qSwc8* affecting SWC under stress condition was located in the vicinity of *PSM377* for conditional relative root length [7] and *qALRR-8* for root length ratio [44] under Al stress, respectively.

Up to now, several genes involved in Al toxicity tolerance have been cloned in rice [20,41,43], while they were all detected by root growth related traits at seedling stage. Through candidate gene analysis of important QTL, we identified 21 candidate genes for 7 important QTL regions associated with up ground traits. For *qSh1*, *LOC_Os01g66250* was the most likely candidate gene because it encodes S-locus-like receptor protein kinase, which was reportedly involved in abiotic stress in rice [45]. Of the three candidate genes for *qSh7*, the most like one was *LOC_Os07g11320* (Alpha-amylase inhibitor/seed allergenic protein), as it was reported to enhance strong early vigor of seedlings to external environment [46]. The most likely candidate genes for *qSdw2* included *LOC_Os02g09220* and *LOC_Os02g09240* even though no haplotype was observed in them. These two genes were all encode cytochrome P450, which was regulated by *ART1* under Al treatment [47]. Of the five candidate genes for *qSdw3a*, *LOC_Os03g47360* (encoding F-box domain containing protein) was the most likely one, which F-Box protein was reported to be expressed under abiotic stress conditions in rice [48]. Of the four candidate genes for *qSdw5*, *LOC_Os05g11730* was most likely candidate because it encodes glycogen synthase kinase 3, which play important roles in development and stress responses in rice [49]. Similarly, *LOC_Os03g27250* was the most likely candidate gene for *qSwc3*, which it also encode F-box protein and was found up expression in cadmium tolerance in *Brassica napus*. For *qSwc8*, the most likely candidate gene *LOC_Os08g03290* encodes glyceraldehyde-3-phosphate dehydrogenase which was involved in oxidative stress through ROS signal pathway [50].

### Importance of above ground traits for evaluating Al toxicity tolerance in rice

Root growth under Al stress was typically used as an indicator to evaluate Al toxicity tolerance. Several visual measuring parameters of root growth were used for QTL mapping for Al toxicity tolerance, such as root length [6,19], root elongation [5,9], relative root length [4,20] and relative root elongation [3,11,18]. Compared with root length, measurement of root elongation is time-consuming and need to use seedlings with uniform root length before beginning of Al stress. In this study, we used 5 d-old rice seedlings exposed to high concentration (1 mM L^-1^) of Al stress under Yoshida solution for 21 days and measured root length and the above ground traits such as SH, SDW and SWC. Using this relative simple method, we identified 16 QTL and 21 candidate genes associated with the above ground traits under Al stress condition. We also confirmed *Nrat1* as a major QTL for Al toxicity tolerance using F_2_ population combined with BSA-seq and linkage mapping methods. Therefore, we think measurement of above ground traits under Al stress is an alternative way to evaluate Al toxicity tolerance in rice, especially in rice breeding for Al toxicity tolerance.

### Integration of GWAS with BSA-seq in QTL mapping and gene identification

QTL mapping is a fundamental approach for realizing the genetic variation of quantitative traits and can provide available markers for marker-assisted selection (MAS) breeding. However, traditional linkage mapping requires screening polymorphic markers based on gel electrophoresis and sometimes sufficient polymorphic markers are not easily available for fine mapping, so it is laborious and low efficient. Moreover, individuals used in traditional linkage mapping were usually derived from biparents, which have low level of genetic variations. This limiting factor for QTL mapping can be exempted by using GWAS combined with natural resources and high density SNP markers [23]. Actually, GWAS could obtain large natural variation and identify a number of significant associations, which promotes fine mapping and gene discovery. This was also illustrated by our GWAS analysis, in which 6 QTL for Al toxicity tolerance were identified to have overlap with the previous reported QTL (Table 1), and 21 candidate genes (Table 2) were inferred. However, the population used in this study was comparatively small and composed exclusively of *indica* accessions. Although intra-subspecific analyses decrease the incidence of false-positive associations resulting from population structure, loci with low MAF or unique to other subpopulations were not detected by GWAS such as *Nrat1* as a major QTL on chromosome 2 in this study, thus some functional alleles probably escaped detection in this *indica* panel [51]. We selected significant SNPs in genes and performed haplotype analysis by non-synonymous SNP in CDS region for candidate genes analysis. There was uncertainty in candidate gene detection for some important QTL such as (*qSdw2* and *qSwc3*) due to absence of haplotype, resulting from low numbers of significant non-synonymous SNPs. Therefore, high SNP density and large population size are strongly recommended for identification of candidate genes through GWAS [30].

**Table 2:** List of 21 candidate genes for 7 important QTL associated with Al toxicity tolerance.

QTL	Candidate genes	Gene annotation
*qSh1*	*LOC_Os01g66100*	gibberellin 20 oxidase 2, putative, expressed
	*LOC_Os01g66250*	S-locus-like receptor protein kinase, putative, expressed
	*LOC_Os01g66360*	2-C-methyl-D-erythritol 4-phosphate cytidylyltransferase, putative, expressed
*qSh7*	*LOC_Os07g11130*	retrotransposon protein, putative, Ty3-gypsy subclass
	*LOC_Os07g11230*	retrotransposon protein, putative, unclassified, expressed
	*LOC_Os07g11320*	RAL1—Seed allergenic protein RA5/RA14/RA17 precursor, expressed
*qSdw2*	*LOC_Os02g09220*	cytochrome P450, putative, expressed
	*LOC_Os02g09240*	cytochrome P450 71D8, putative, expressed
*qSdw3a*	*LOC_Os03g47240*	expressed protein
	*LOC_Os03g47310*	transposon protein, putative, CACTA, En/Spm sub-class, expressed
	*LOC_Os03g47330*	transposon protein, putative, CACTA, En/Spm sub-class
	*LOC_Os03g47360*	expressed protein
	*LOC_Os03g47370*	LTPL95—Protease inhibitor/seed storage/LTP family protein precursor, putative, expressed
*qSdw5*	*LOC_Os05g11460*	expressed protein
	*LOC_Os05g11630*	expressed protein
	*LOC_Os05g11660*	expressed protein
	*LOC_Os05g11730*	CGMC_GSK.7—CGMC includes CDA, MAPK, GSK3, and CLKC kinases, expressed
*qSwc3*	*LOC_Os03g27230*	phospho-2-dehydro-3-deoxyheptonate aldolase, chloroplast precursor, putative, expressed
	*LOC_Os03g27250*	OsFBO14—F-box and other domain containing protein, expressed
*qSwc8*	*LOC_Os08g03180*	retrotransposon protein, putative, unclassified, expressed
	*LOC_Os08g03290*	glyceraldehyde-3-phosphate dehydrogenase, putative, expressed

Alternatively, BSA-seq is another quick and efficient method for QTL mapping, which is a combination of bulked-segregant analysis (BSA) and whole genome re-sequencing by NGS technologies. Based on association mapping results in the present study, we sequenced two extremely DNA pools each composed of 20 F_2_ individuals derived from the two accessions with opposite phenotype under Al stress and identified the major gene *Nrat1* for Al toxicity tolerance by linkage analysis of extremely selective F_2_ individuals using 11 KASP markers developed from the region of 0.16–2.79 Mb on chromosome 2. The region of 0.16–2.79 Mb harboring *Nrat1* identified by BSA-seq and linkage analysis is different from that of 4.61–4.86 Mb harboring the major QTL identified by GWAS, probably resulting from the fact that the accession CC105 (PR) has two favorable alleles for improving Al toxicity tolerance at the above two loci on chromosome 2, and the locus of *Nrat1* has much larger phenotypic effect than that of the major QTL due to the super susceptible allele from the CC180 (PS), which resulted in masking effect of the major QTL when BSA-seq was applied.

Comparing with screening conventional markers in candidate QTL region, KASP markers have many advantages, such as simple, efficient and accuracy as demonstrated in QTL mapping and MAS-based pyramiding of drought and low nitrogen tolerances in rice [52]. Taken together, GWAS can efficiently screen important materials with targeted traits and identify significant QTL regions or candidate genes. Then discover the candidate genes responsible for the targeted traits by BAS-seq analysis and linkage analysis as shown in this study. Our results showed that integration of GWAS and BSA-seq greatly improved the efficiency in identifying favorable genes contained in germplasms.

### SNP variation in *Nrat1* affected Al toxicity tolerance in rice

Rice cultivation has been sustainable for thousands of years in Asia, so few allele variations affecting Al toxicity tolerance can be found in different varieties because of the domestication and natural selection. *Nrat1* play an important role in basal Al toxicity tolerance mechanisms in rice and was identified by different populations [20,41,53]. Each of the mutant amino acids in *Nrat1* is of equal importance for full *Nrat1* Al transport function [42]. Xia et al. compared nine rice varieties, no consistent trend between promoter sequence and *Nrat1* expression, and no trend was found between amino acid sequence and Al toxicity tolerance [29]. In another research, three non-synonymous SNPs were found among the nine highly susceptible rice accessions, but none SNP alone was predictive of Al toxicity tolerance [20], indicating that the functional site in *Nrat1* was depended on specific genetic background. Unlike previous studies on the analysis of *Nrat1*, CC180 (PS), a super sensitive accession, had a new natural variation in the coding region of *Nrat1*, which caused big difference in Al toxicity tolerance between CC105 (PR) and PS and explained 88.7% phenotypic variation, suggesting the novel allele is important in genetic study or rice breeding for Al toxicity tolerance. This SNP was further confirmed by amplifying the sequence of coding region and promoter region in the two parents using PCR. However, there was no SNP variation in this site found in 3K-Rice Genome Project, suggesting it was a minor allele in *Nrat1*. Thus, it is also important to examine minor alleles in rice breeding when using natural gene resources.

## Conclusion

A total of 21 QTL for Al toxicity tolerance at seedling stage were detected through GWAS and 8 most like candidate genes for 7 important QTL were identified according to haplotype analysis and gene functional annotation. *Nrat1* (*LOC_Os02g03900*) is responsible for Al toxicity tolerance located in the region of 0.16–2.79 Mb on chromosome 2 detected by BSA-seq and linkage analyses. Comparison of cDNA sequence in *Nrat1* between PR and PS, a new non-synonymous SNP variation was observed, which resulted in an amino-acid substitution from Ala (A) in PR to Asp (D) in PS. Haplotype analysis of *Nrat1* indicated that minor allele variations in *aus* and *indica* subpopulation decreased Al toxicity tolerance in rice. Our results indicated that minor alleles are important for QTL mapping and application in rice breeding when natural gene resources are used.

## Supporting information

S1 Fig Correlations of all measured traits under control (upper triangular) and Al stress (lower triangular) conditions. The values on the principal diagonal were correlation coefficients of the same trait between control and stress conditions. Ellipses obliquing to right indicated positive correlations. The values without glyphs indicated insignificant at 0.05.
(JPG)

S2 Fig Expression pattern of *Nrat1* in PR and PS. (JPG)

S1 File Quantile-Quantile plot comparing p-values for the mixed model. Colored solid lines of the observed ordered -log10 (*p*-value) on the Y-axis vs expected -log10 (*p*-value) on the X-axis.
(PPTX)

S2 File All primers sequence used in this study. (XLSX)

S1 Table ANOVA results of the measured traits under Al toxicity for 211 *indica* accessions. (DOCX)

S2 Table ANOVA results of all measured traits under control and Al toxicity conditions for 211 indica accessions. (DOCX)

S3 Table Statistic of sequencing results. (DOCX)

S4 Table List of 6 SNP with ΔSNP index equal to 1 and neared genes. (DOCX)
