# Supplementary figures and images for: Genome-wide association mapping of aluminum toxicity tolerance and fine mapping of a candidate gene for Nrat1 in rice
Source: PLoS One. 2018 Jun 12;13(6):e0198589. doi: 10.1371/journal.pone.0198589 (PMC5997306; doi:10.1371/journal.pone.0198589)

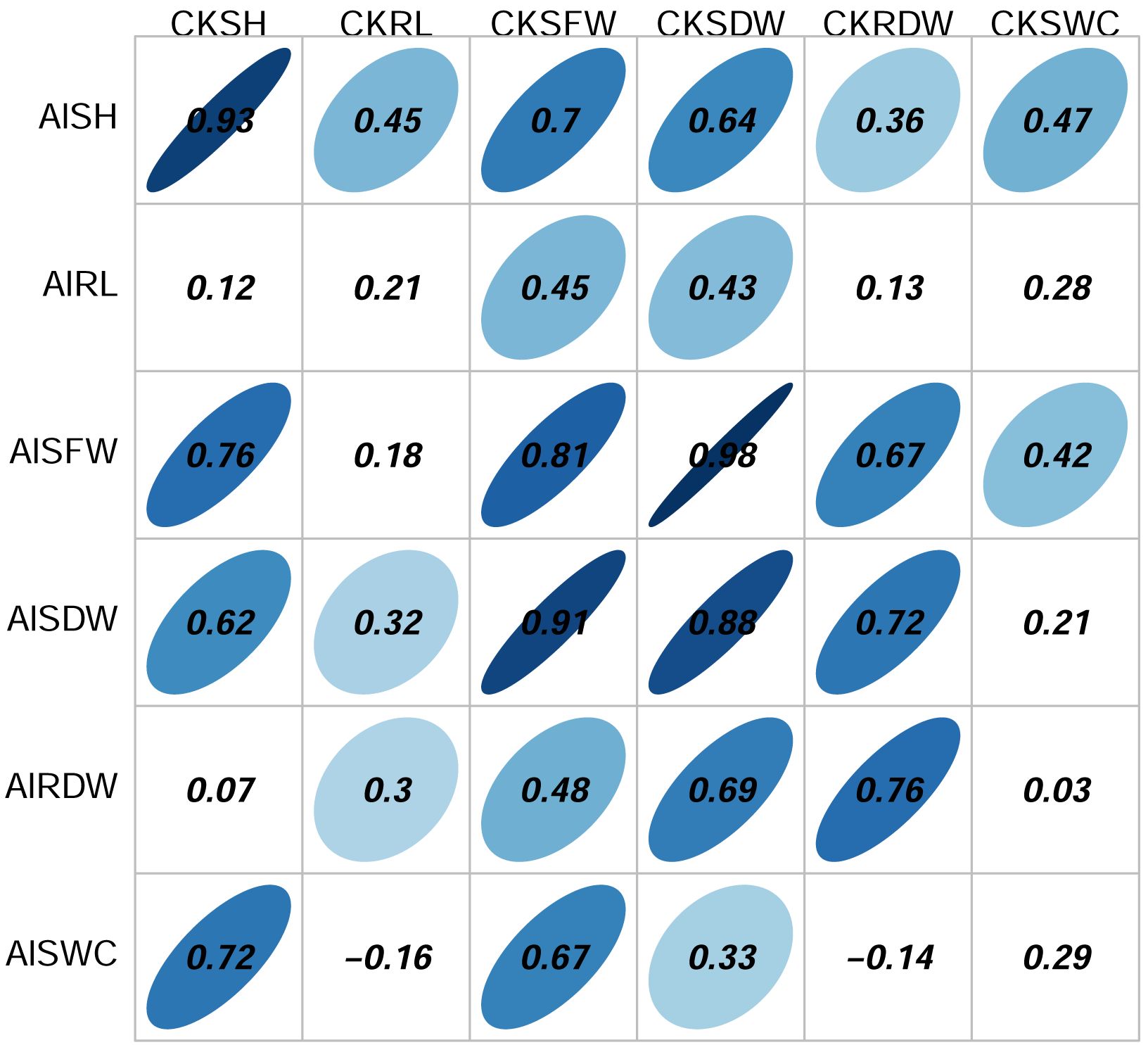

Supplement: S1 Fig — The values on the principal diagonal were correlation coefficients of the same trait between control and stress conditions. Ellipses obliquing to right indicated positive correlations. The values without glyphs indicated insignificant at 0.05. (JPG) [file pone.0198589.s001.jpg]

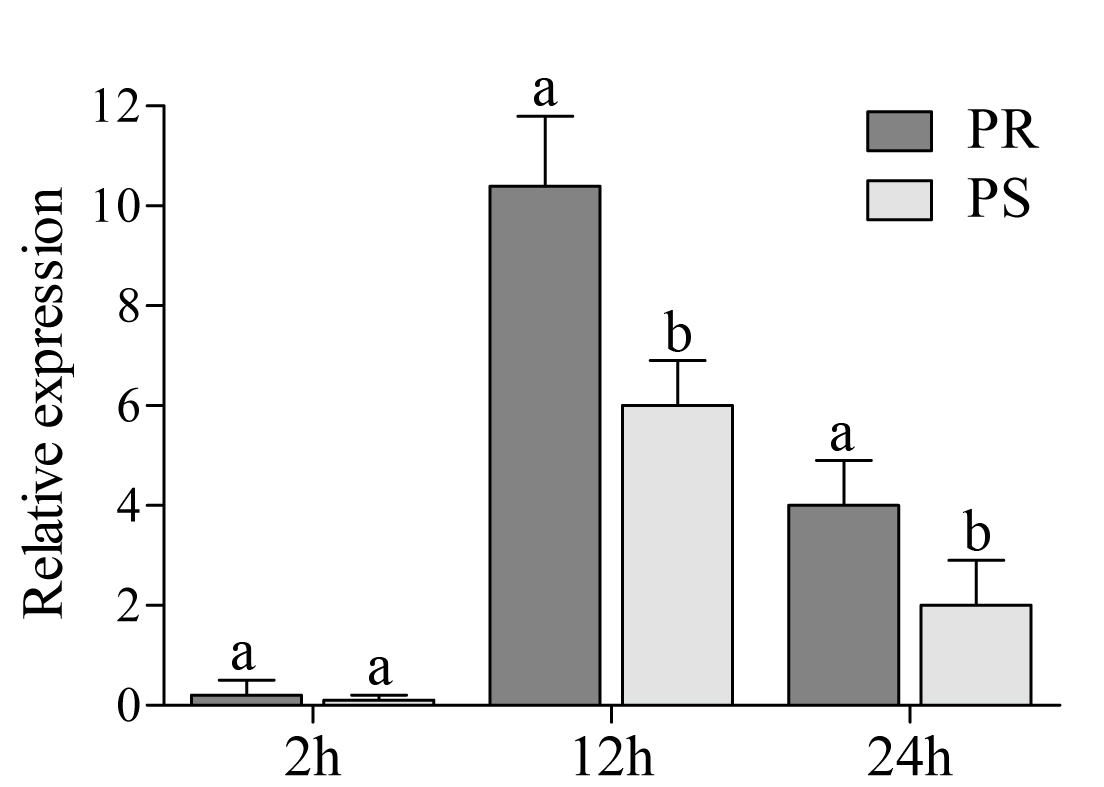

Supplement: S2 Fig — (JPG) [file pone.0198589.s002.jpg]
