# Supplementary material for: Genome-wide association mapping of aluminum toxicity tolerance and fine mapping of a candidate gene for Nrat1 in rice
Source: PLoS One. 2018 Jun 12;13(6):e0198589. doi: 10.1371/journal.pone.0198589 (PMC5997306; doi:10.1371/journal.pone.0198589)

## Slide 1
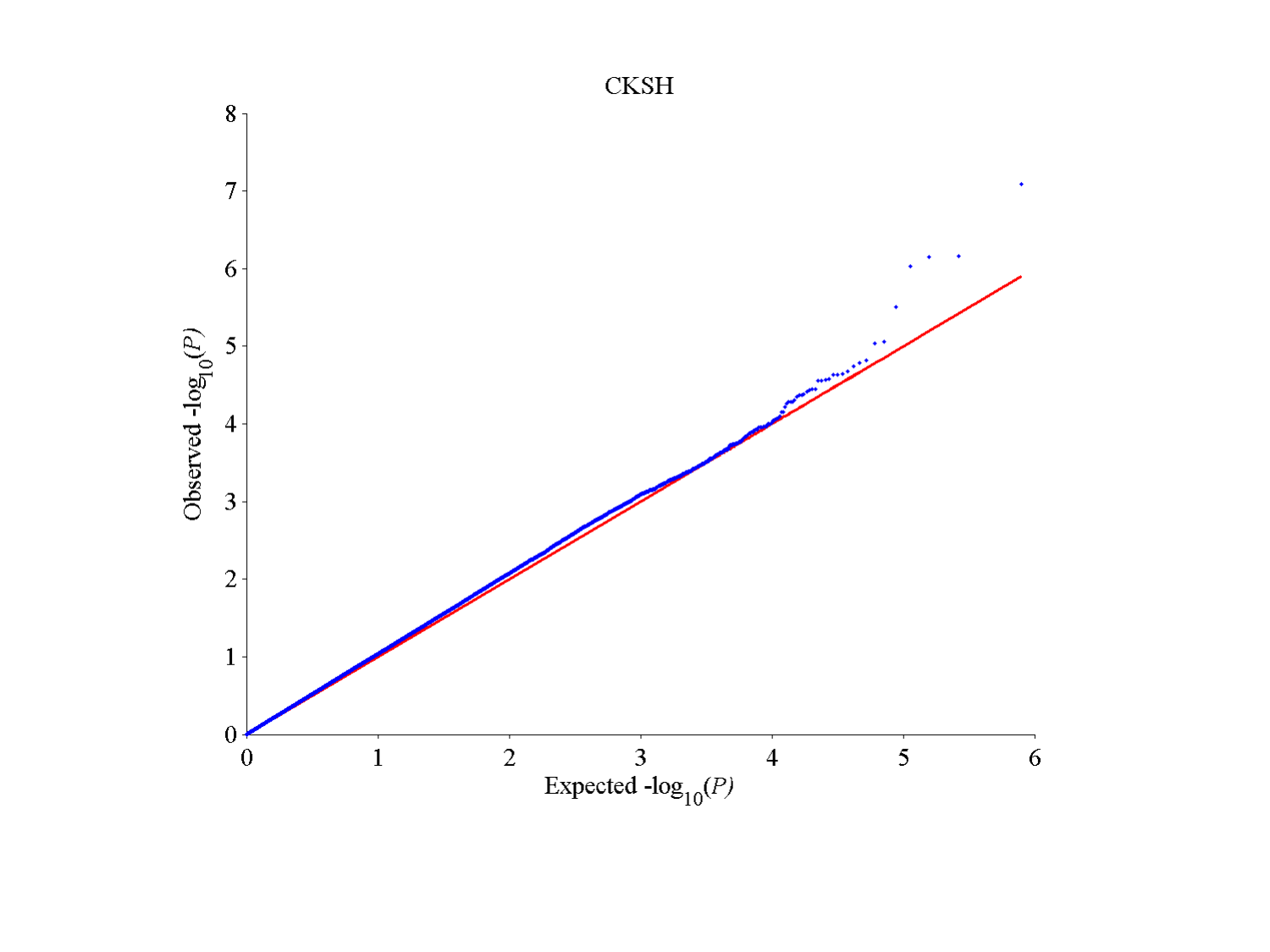

## Slide 2
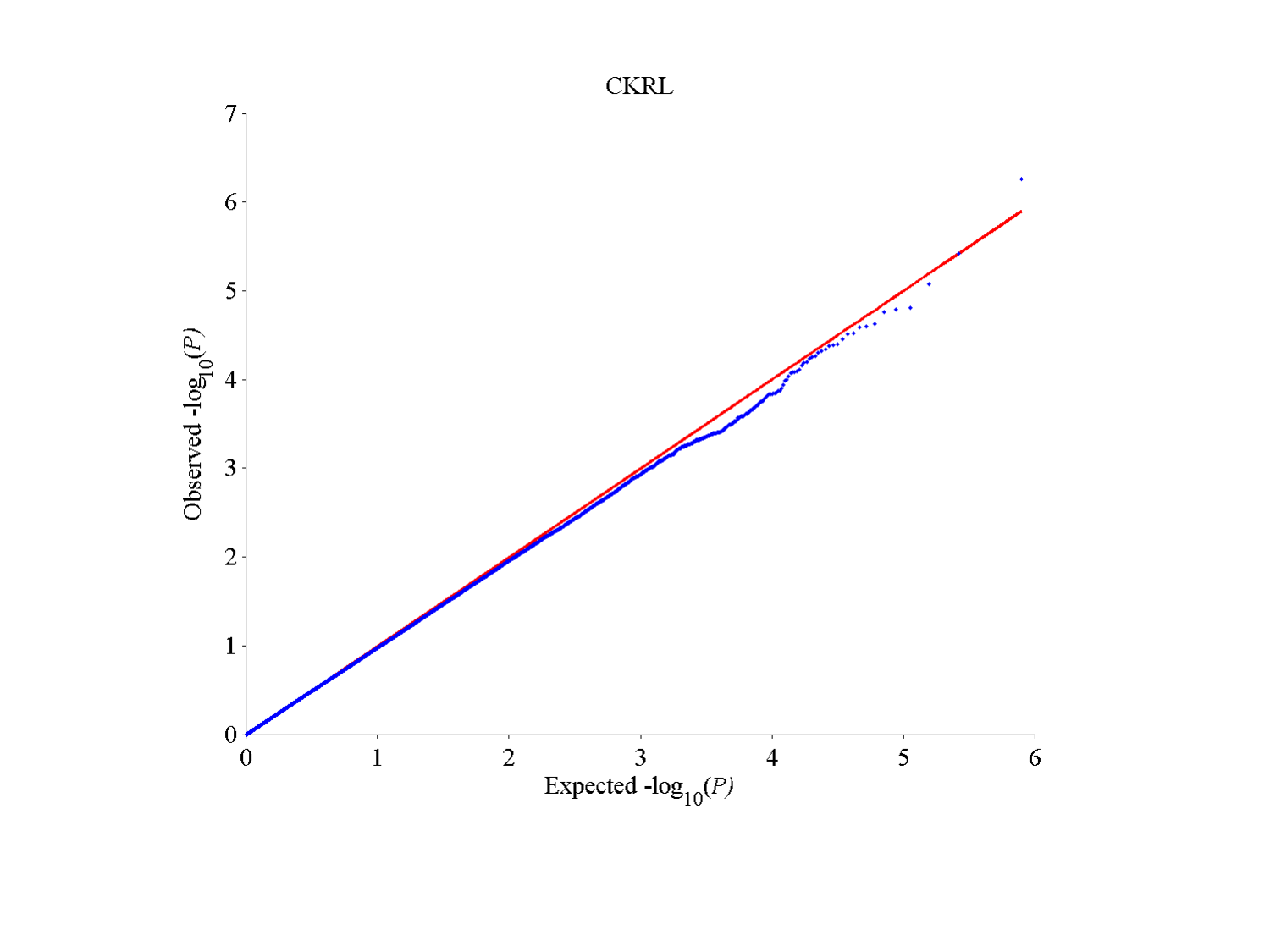

## Slide 3
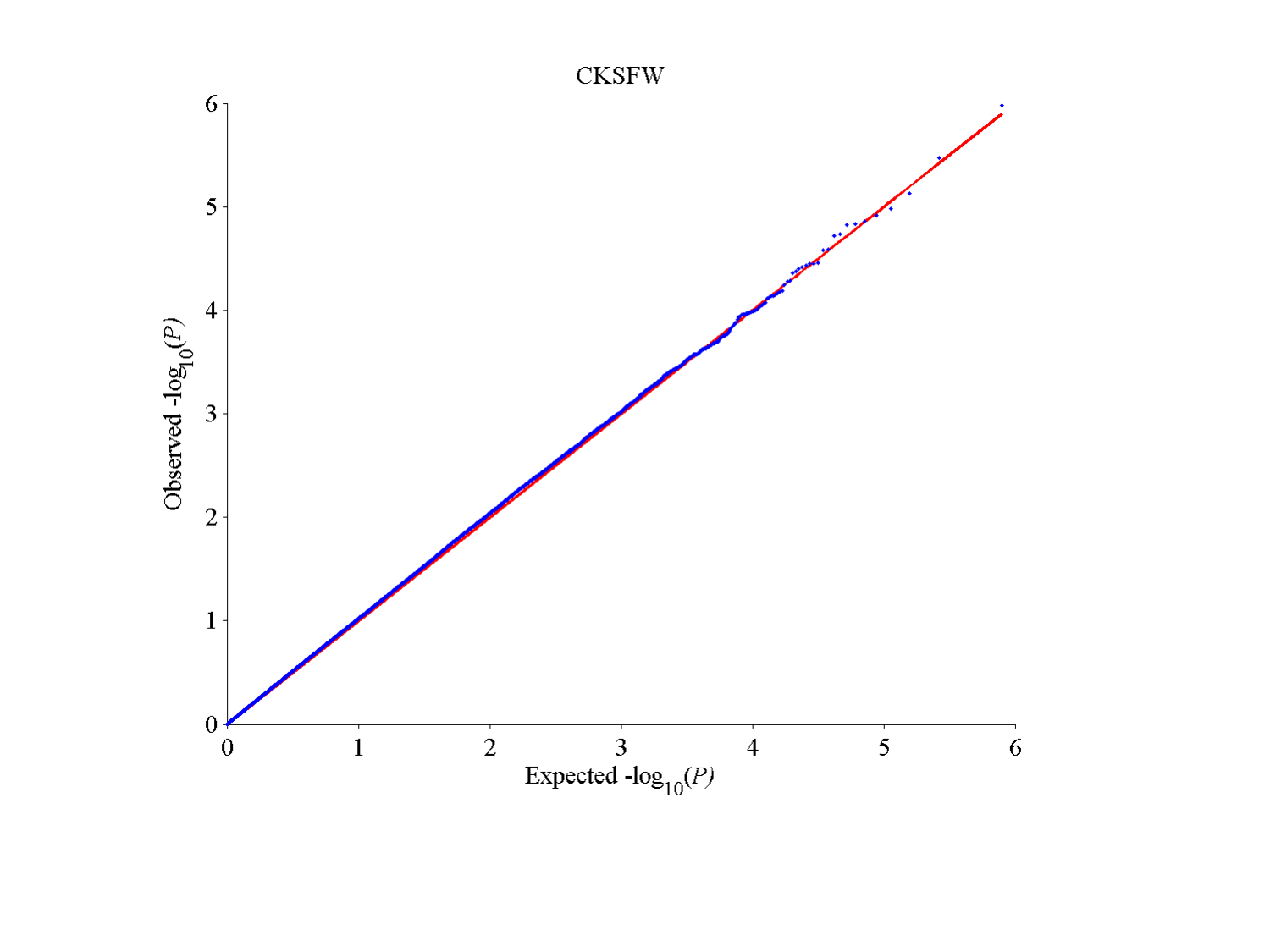

## Slide 4
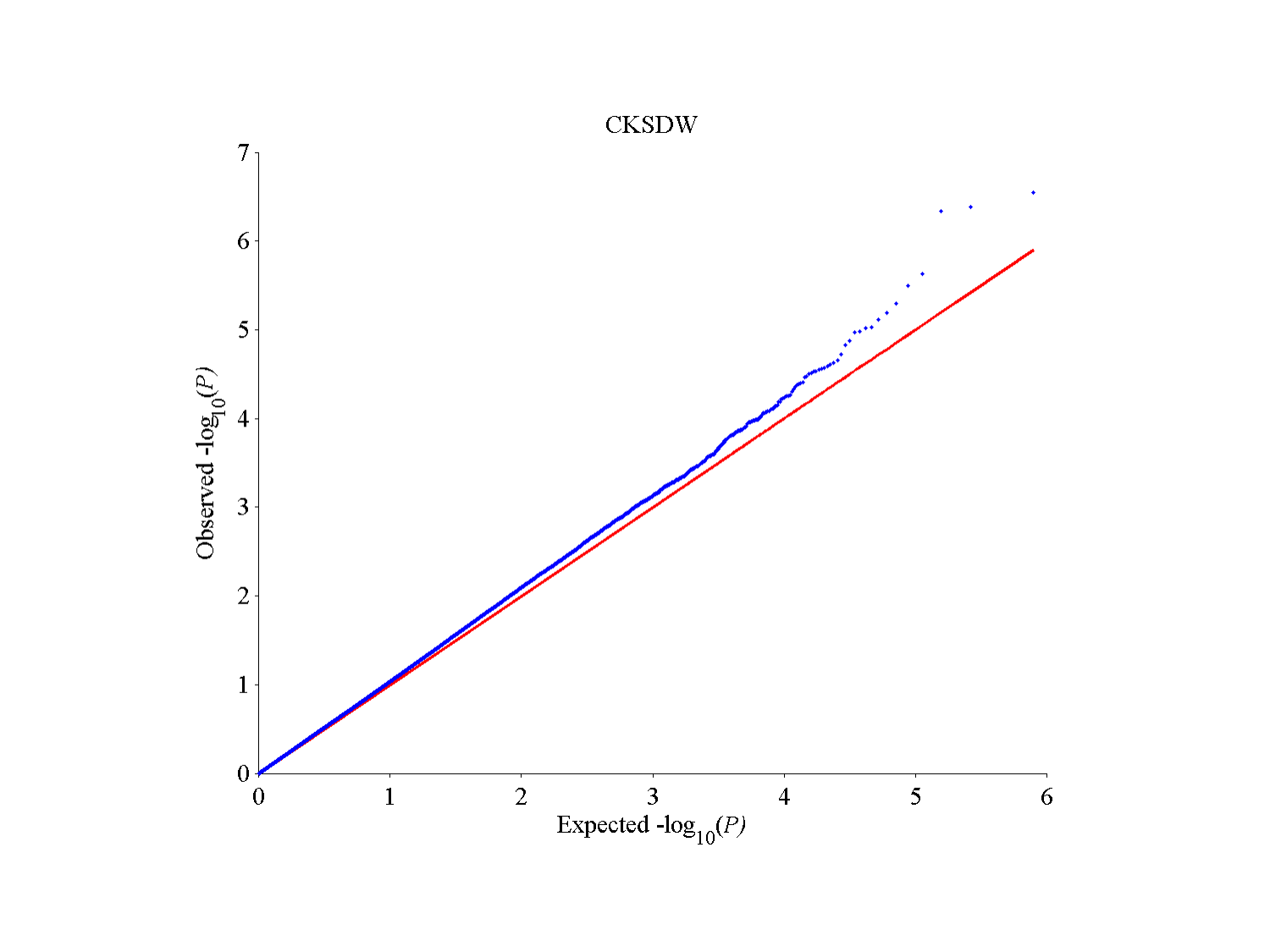

## Slide 5
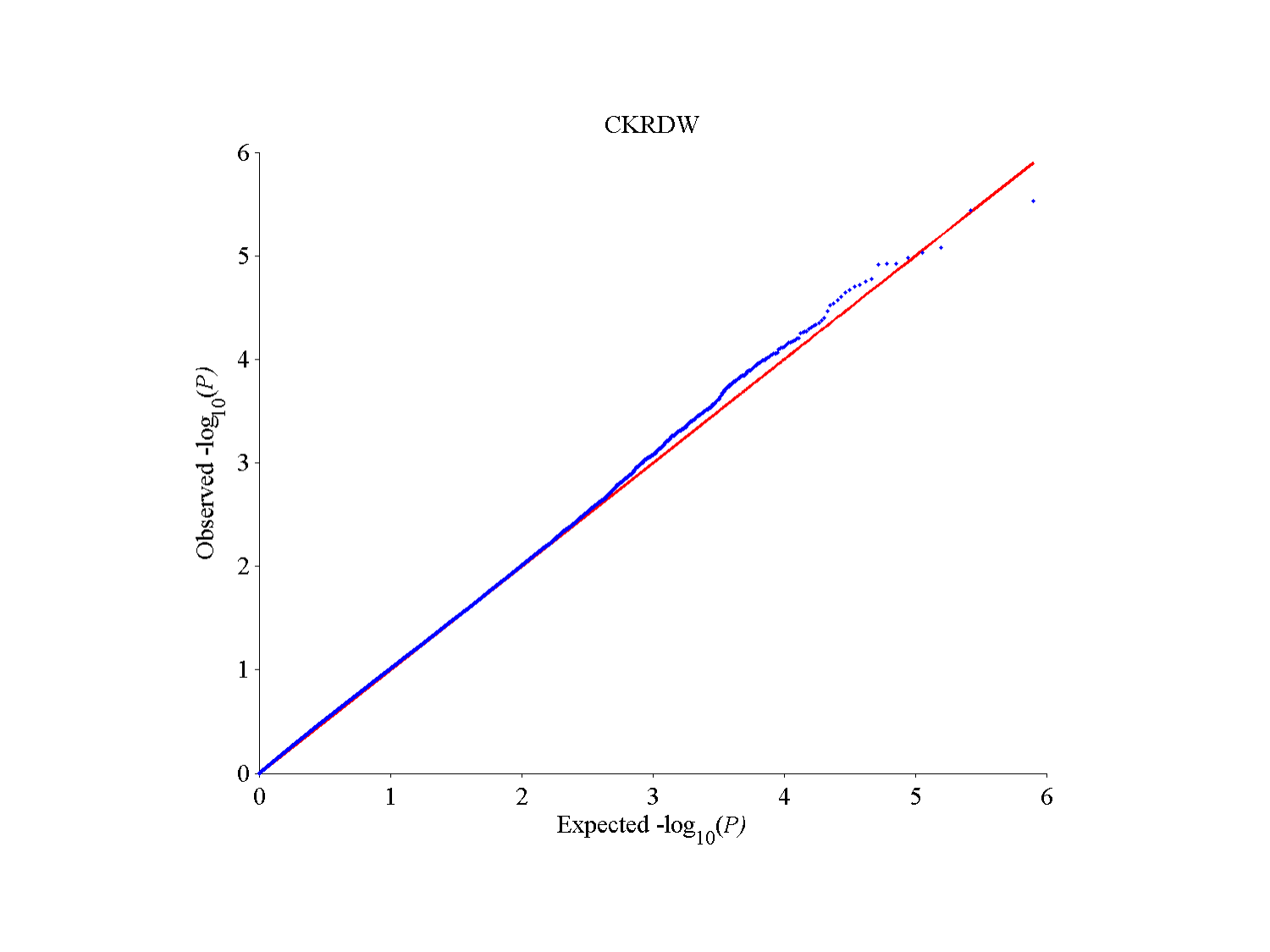

## Slide 6
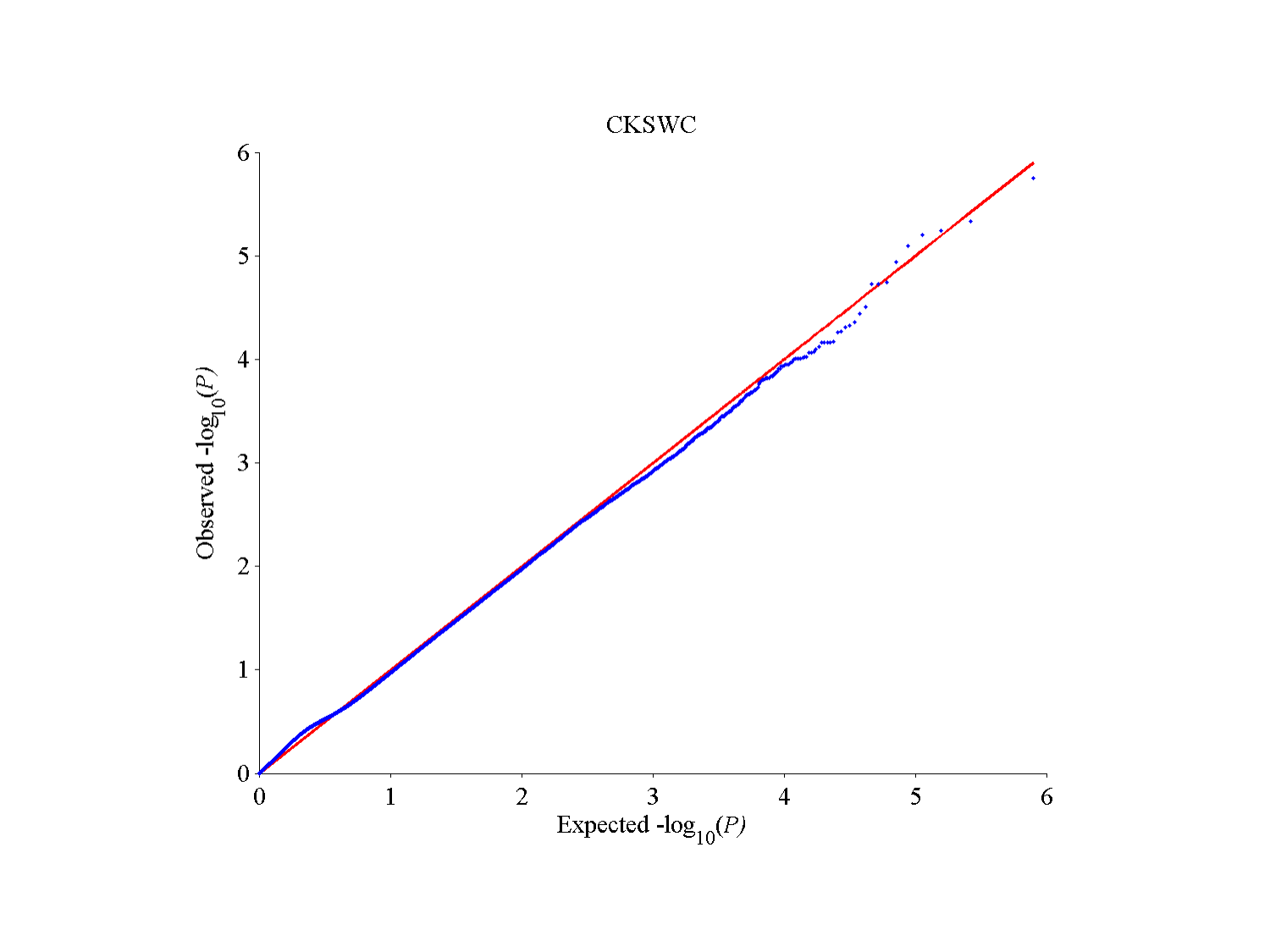

## Slide 7
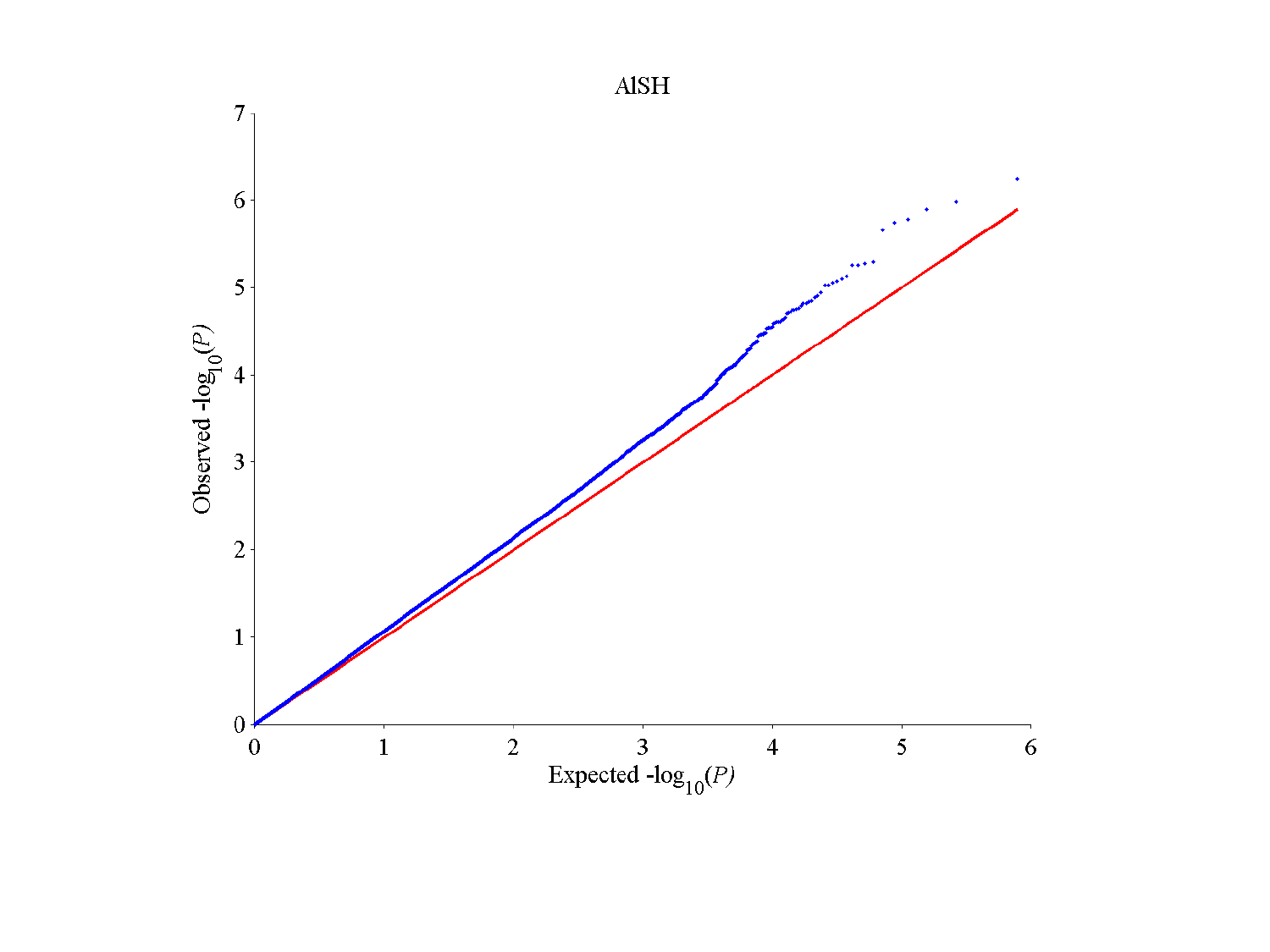

## Slide 8
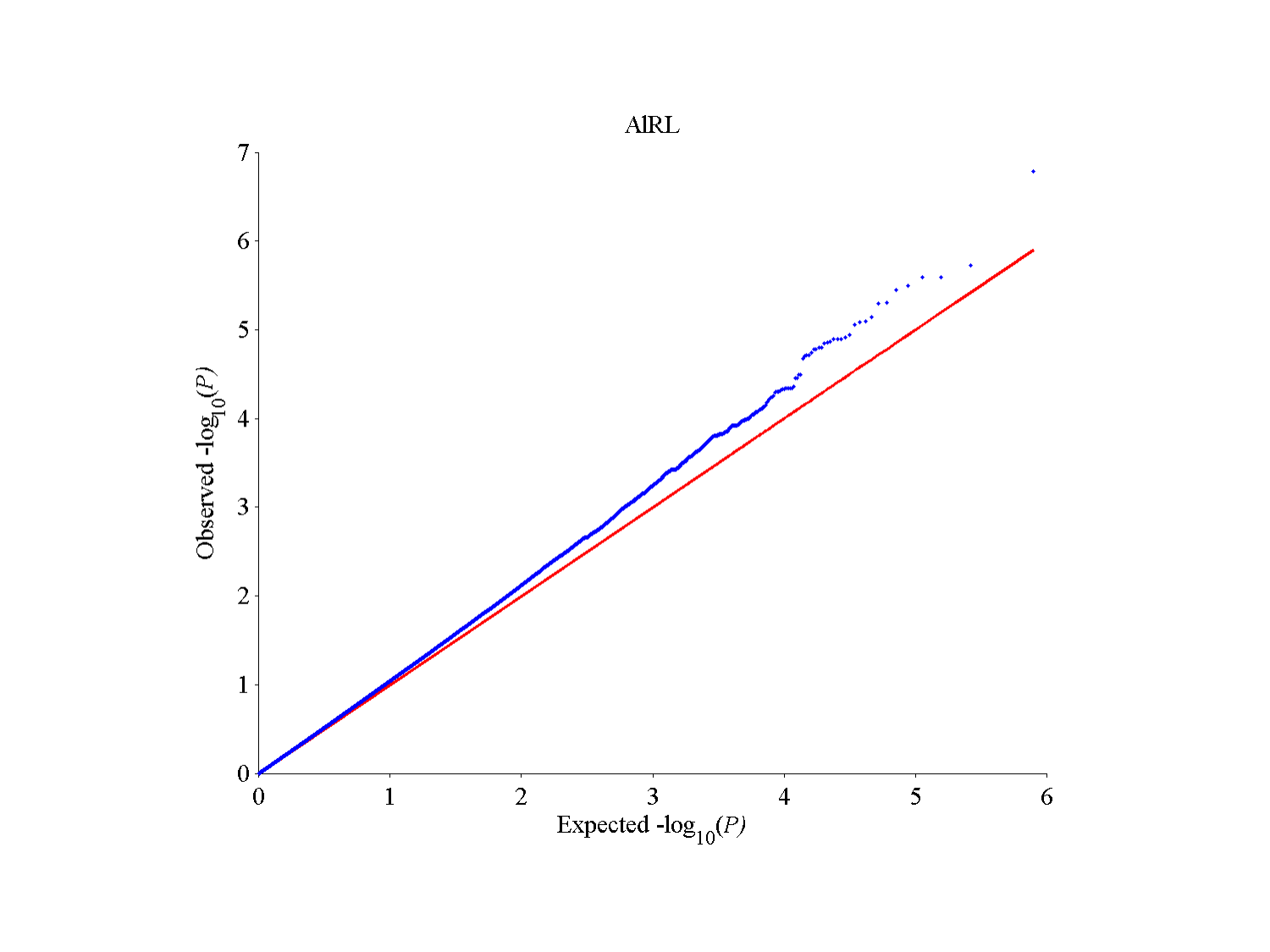

## Slide 9
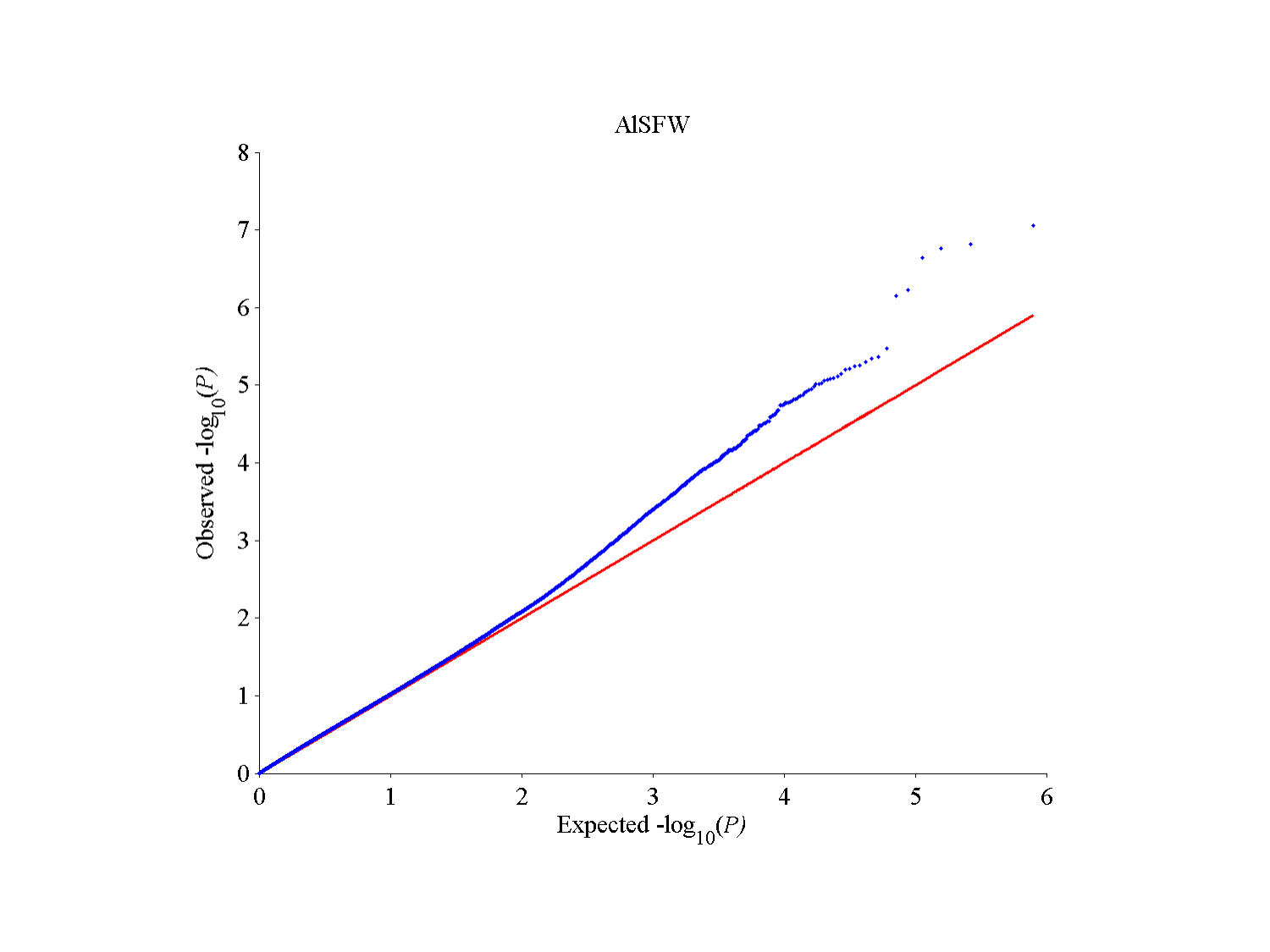

## Slide 10
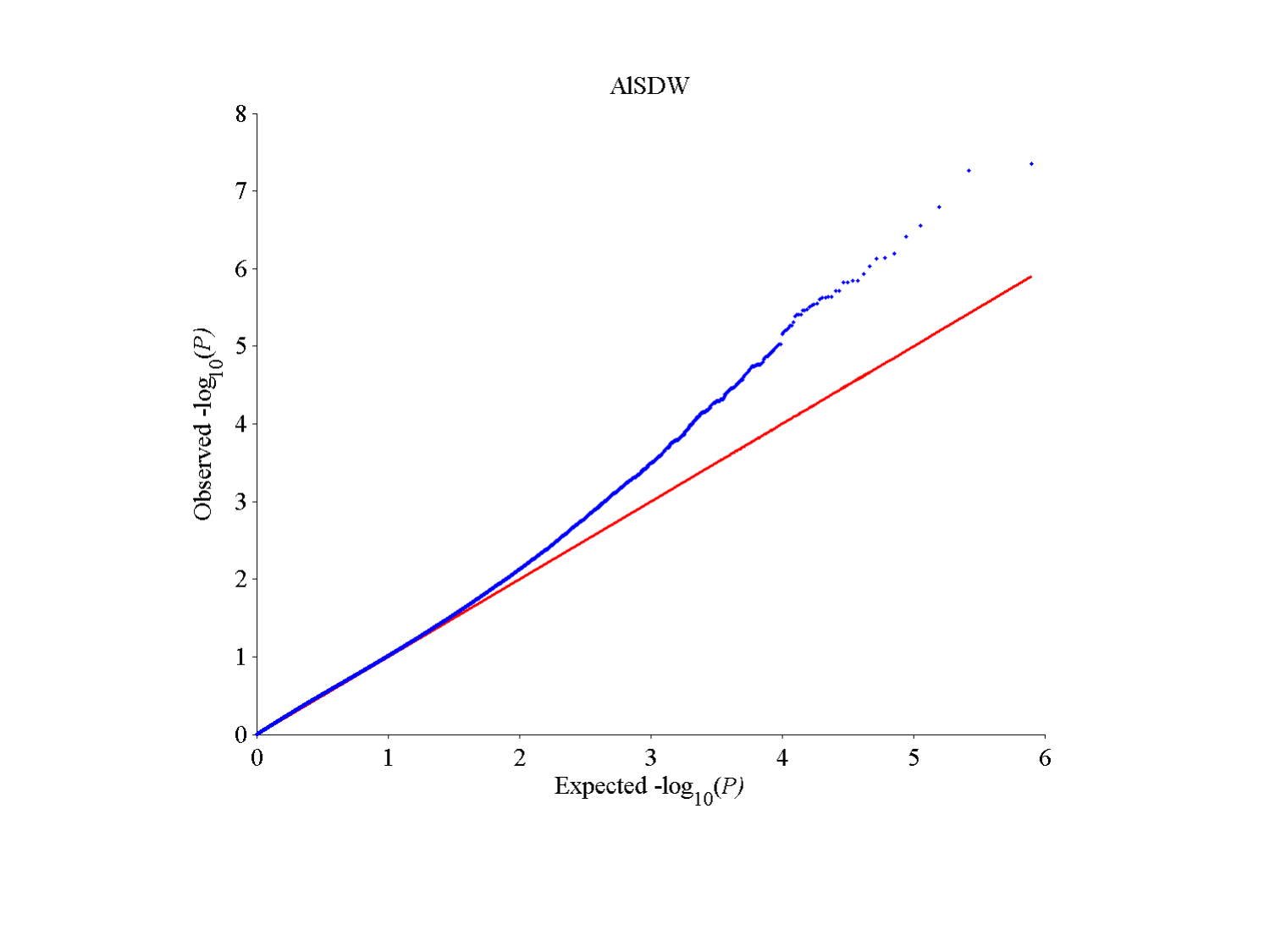

## Slide 11
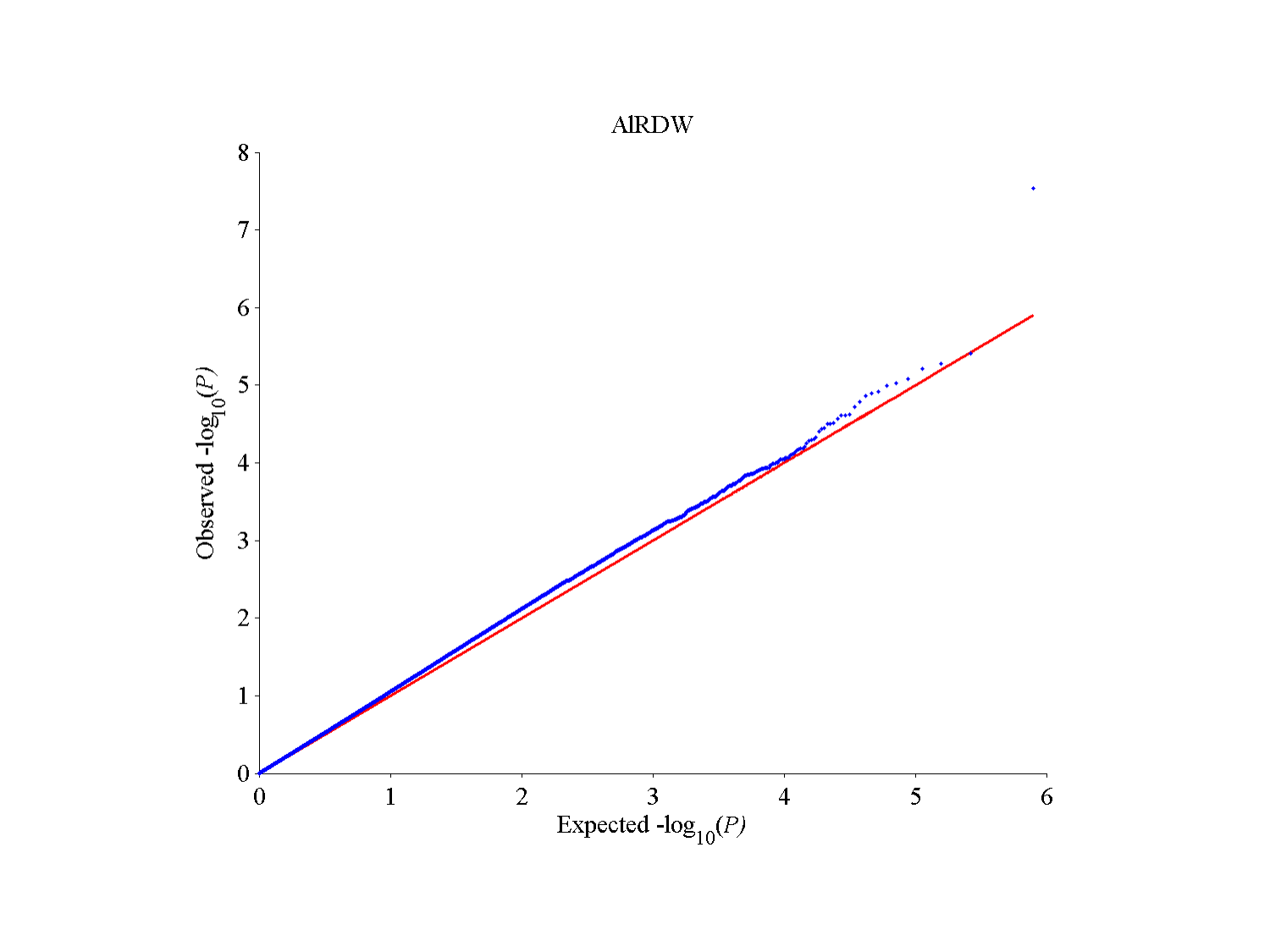

## Slide 12
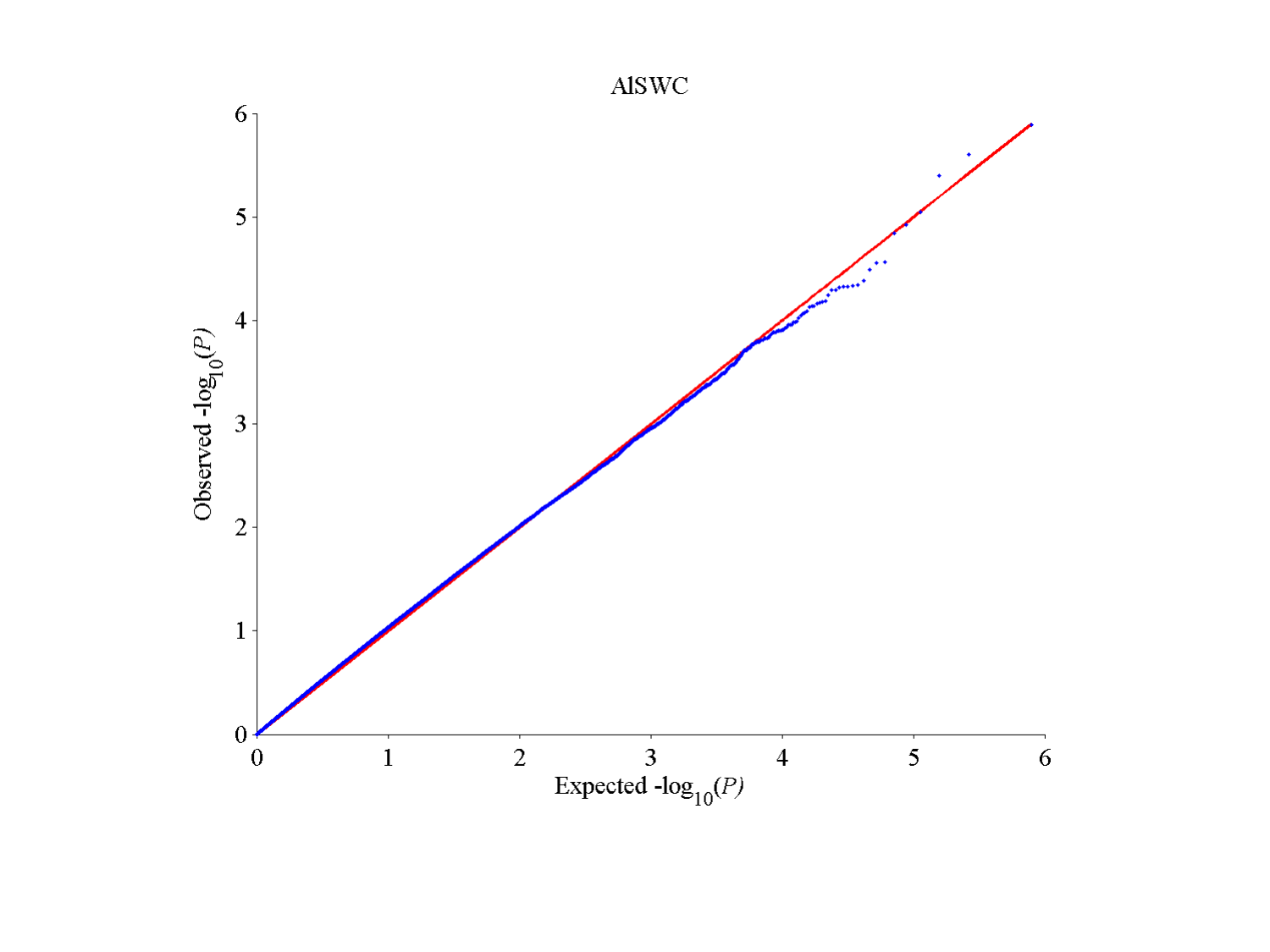

## Slide 13
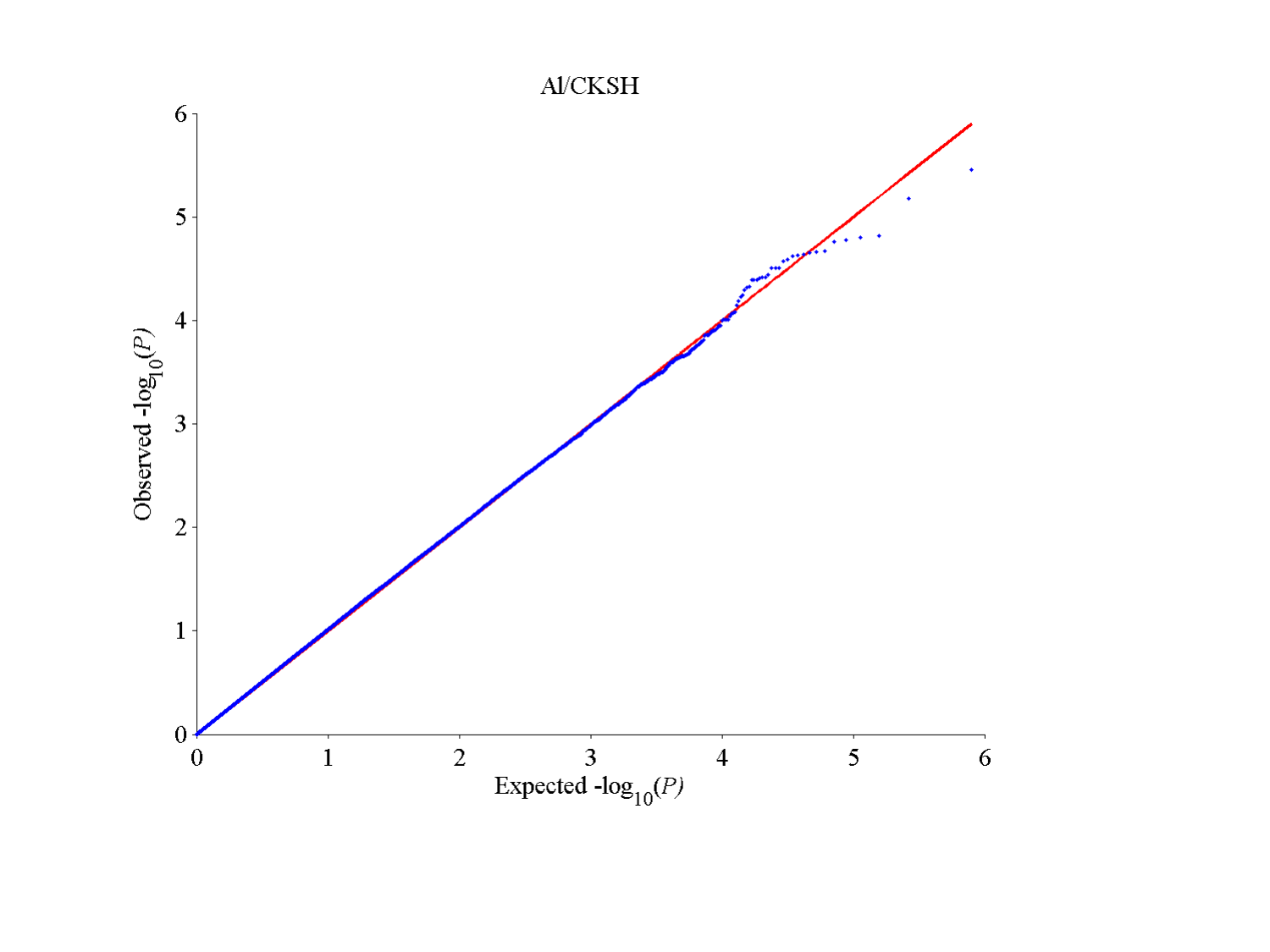

## Slide 14
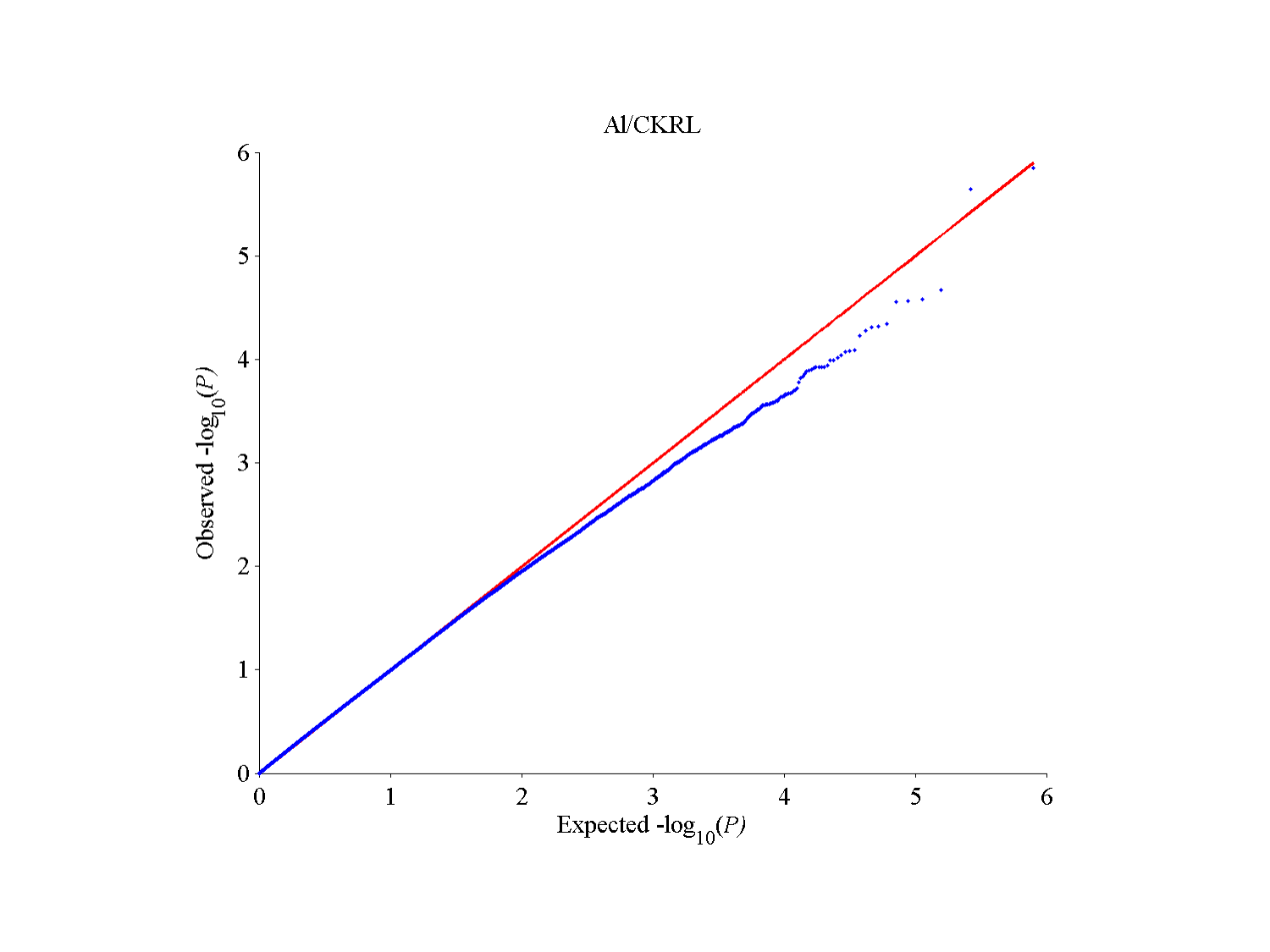

## Slide 15
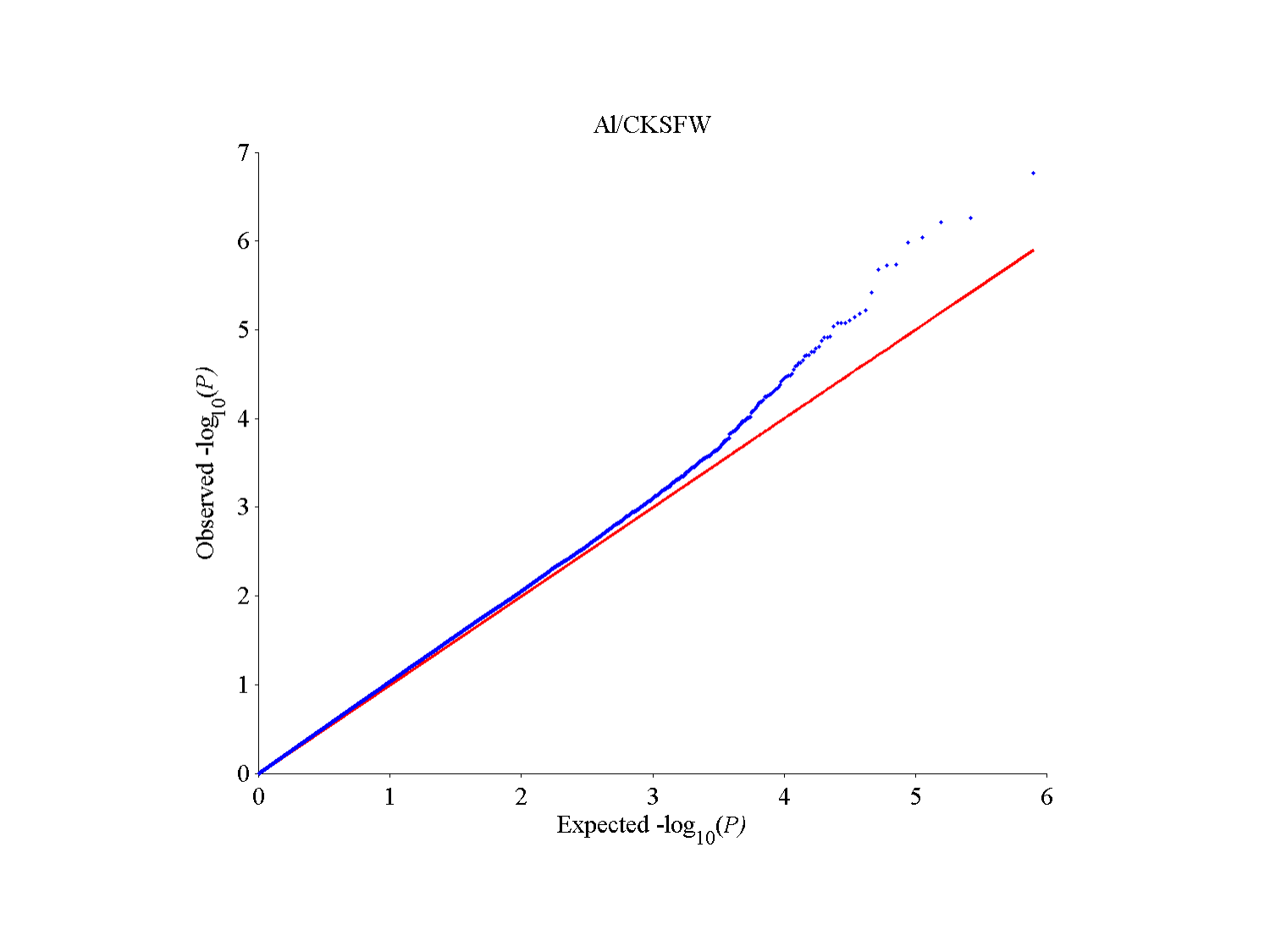

## Slide 16
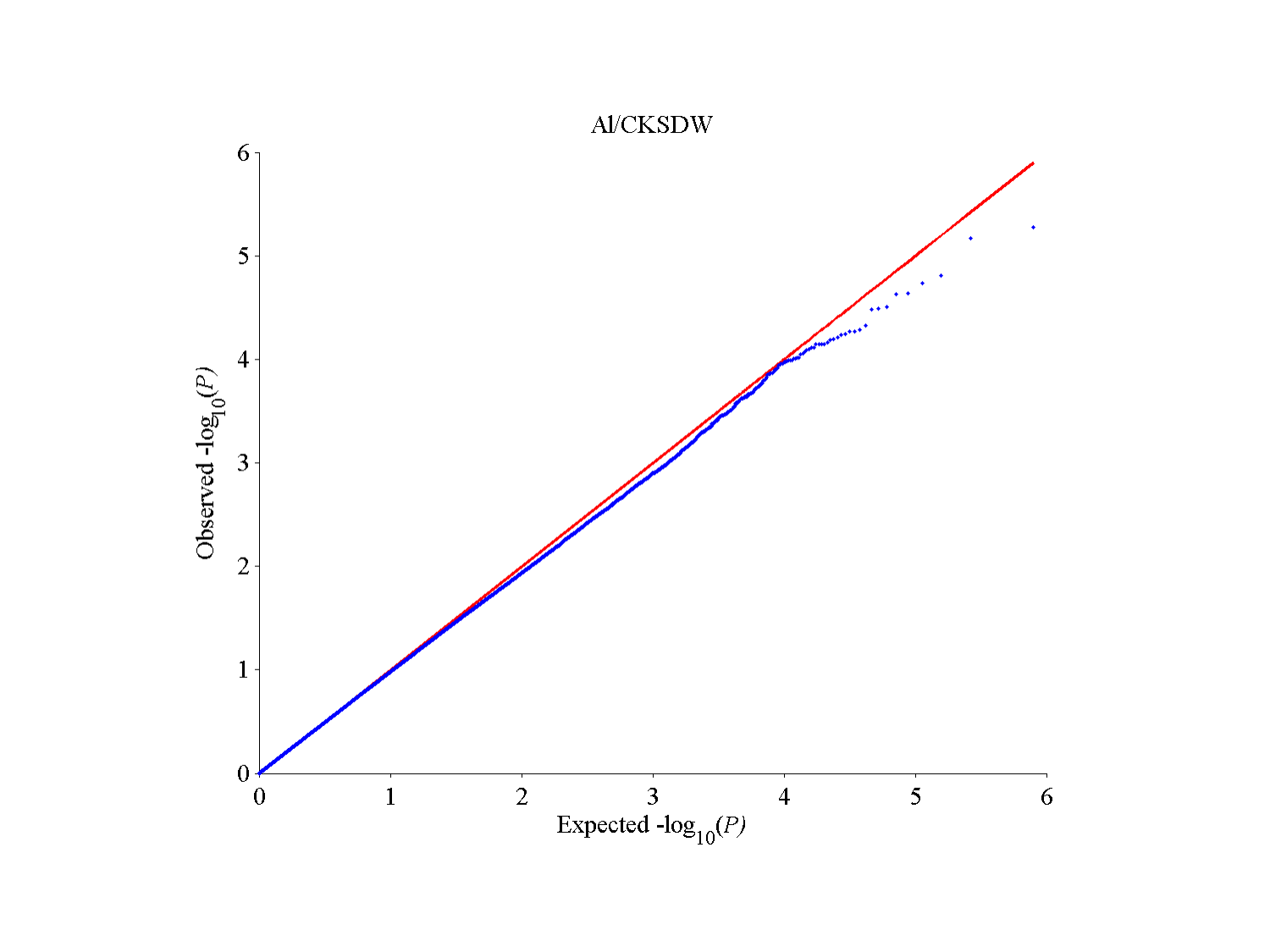

## Slide 17
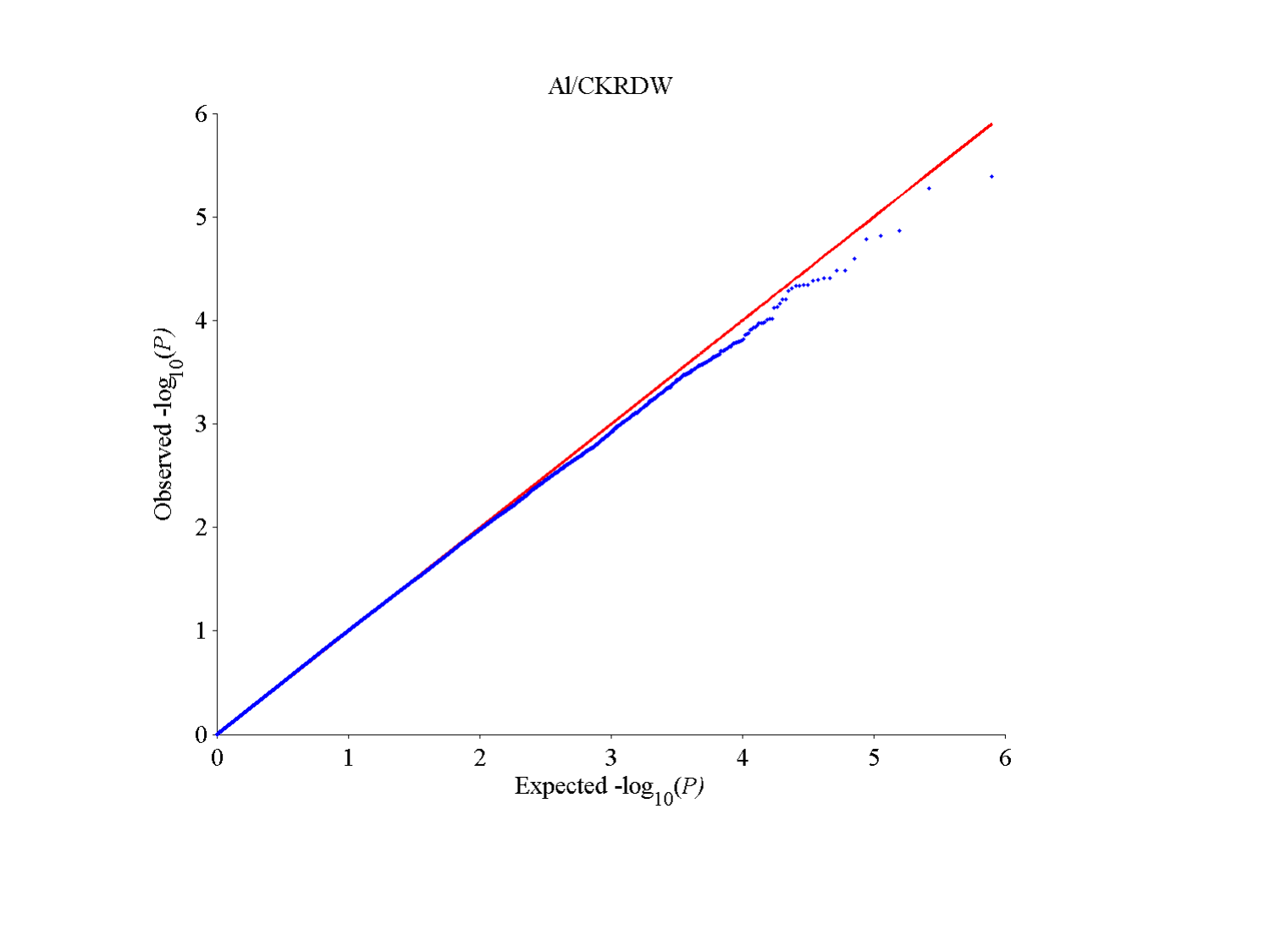

## Slide 18
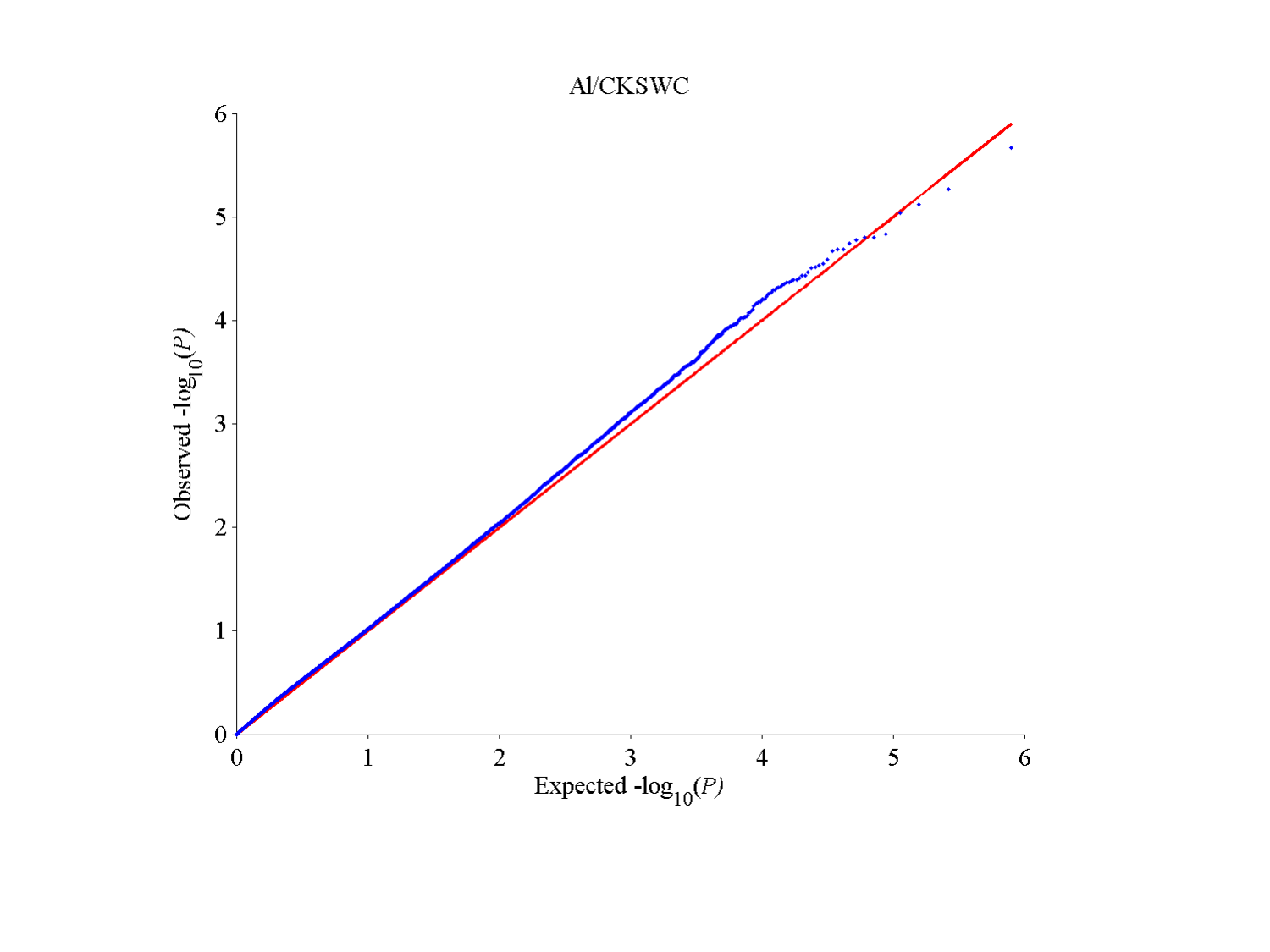

Supplement: S1 File — Colored solid lines of the observed ordered -log10 (p-value) on the Y-axis vs expected -log10 (p-value) on the X-axis. (PPTX) [file pone.0198589.s003.pptx]
