# Supplementary material for: Genome-wide association mapping of aluminum toxicity tolerance and fine mapping of a candidate gene for Nrat1 in rice
Source: PLoS One. 2018 Jun 12;13(6):e0198589. doi: 10.1371/journal.pone.0198589 (PMC5997306; doi:10.1371/journal.pone.0198589)
Supplement: S1 Table — (DOCX) [file pone.0198589.s005.docx]

**S1 Table. ANOVA results of all measured traits under control and Al toxicity conditions for 211 *indica* accessions**

| **Trait** | **Source of variation** | ***df*** | ***SS*** | ***MS*** | ***F*** | ***P-* value** | ***R*^2^ (%)** |
| --- | --- | --- | --- | --- | --- | --- | --- |
| SH | Environment | 1 | 4800.85 | 4800.85 | 617.89 | <.0001 | 96.6 |
|  | Genotype | 208 | 41739.99 | 200.67 | 25.83 | <.0001 |  |
|  | Error | 208 | 1616.10 | 7.77 |  |  |  |
| RL | Environment | 1 | 1930.49 | 1930.49 | 359.67 | <.0001 | 75.6 |
|  | Genotype | 208 | 1528.36 | 7.35 | 1.37 | 0.012 |  |
|  | Error | 208 | 1116.43 | 5.37 |  |  |  |
| SFW | Environment | 1 | 3517135.51 | 3517135.51 | 754.37 | <.0001 | 92.8 |
|  | Genotype | 208 | 8958523.85 | 43069.83 | 9.24 | <.0001 |  |
|  | Error | 208 | 969768.36 | 4662.35 |  |  |  |
| SDW | Environment | 1 | 23527.73 | 23527.73 | 460.16 | <.0001 | 94 |
|  | Genotype | 208 | 143330.55 | 689.09 | 13.48 | <.0001 |  |
|  | Error | 208 | 10634.94 | 51.13 |  |  |  |
| RDW | Environment | 1 | 1305.45 | 1305.45 | 467.59 | <.0001 | 90.6 |
|  | Genotype | 208 | 4284.27 | 20.60 | 7.38 | <.0001 |  |
|  | Error | 208 | 580.71 | 2.79 |  |  |  |
| SWC | Environment | 1 | 1420.47 | 1420.47 | 376.67 | <.0001 | 76.6 |
|  | Genotype | 208 | 1140.77 | 5.48 | 1.45 | 0.0036 |  |
|  | Error | 208 | 784.40 | 3.77 |  |  |  |
