# Supplementary material for: Genome-wide association mapping of aluminum toxicity tolerance and fine mapping of a candidate gene for Nrat1 in rice
Source: PLoS One. 2018 Jun 12;13(6):e0198589. doi: 10.1371/journal.pone.0198589 (PMC5997306; doi:10.1371/journal.pone.0198589)
Supplement: S2 Table — (DOCX) [file pone.0198589.s006.docx]

**S2 Table. ANOVA results of the measured traits under Al toxicity for 211 *indica* accessions**

| **Trait** | **Source of variation** | ***df*** | ***SS*** | ***MS*** | ***F*** | ***P-* value** | ***R*^2 (^%)** |
| --- | --- | --- | --- | --- | --- | --- | --- |
| CKSH | Genotype | 208 | 38600.32 | 185.58 | 46.6 | <.0001 | 97.9 |
|  | rep | 1 | 0.85 | 0.85 | 0.2 | 0.644 |  |
|  | Error | 208 | 827.89 | 3.98 |  |  |  |
| CKRL | Genotype | 208 | 4505.27 | 21.66 | 4.5 | <.0001 | 81.7 |
|  | rep | 1 | 14.28 | 14.28 | 3.0 | 0.0857 |  |
|  | Error | 208 | 996.35 | 4.79 |  |  |  |
| CKSFW | Genotype | 208 | 11483806.21 | 55210.61 | 25.9 | <.0001 | 95.9 |
|  | rep | 1 | 49397.58 | 49397.58 | 23.2 | <.0001 |  |
|  | Error | 208 | 443568.42 | 2132.54 |  |  |  |
| CKSDW | Genotype | 208 | 190227.16 | 914.55 | 22.6 | <.0001 | 95.7 |
|  | rep | 1 | 59.37 | 59.37 | 1.5 | 0.2274 |  |
|  | Error | 208 | 8425.61 | 40.51 |  |  |  |
| CKRDW | Genotype | 208 | 5892.38 | 28.33 | 14.5 | <.0001 | 90.0 |
|  | rep | 1 | 251.52 | 251.52 | 128.9 | <.0001 |  |
|  | Error | 208 | 405.97 | 1.95 |  |  |  |
| CKSWC | Genotype | 208 | 298629.54 | 1435.72 | 5155.3 | <.0001 | 99.9 |
|  | rep | 1 | 47.51 | 47.51 | 170.6 | <.0001 |  |
|  | Error | 208 | 57.93 | 0.28 |  |  |  |
| AlSH | Genotype | 208 | 43401.85 | 208.66 | 111.0 | <.0001 | 99.1 |
|  | rep | 1 | 4.02 | 4.02 | 2.1 | 0.145 |  |
|  | Error | 208 | 391.17 | 1.88 |  |  |  |
| AlRL | Genotype | 208 | 1547.90 | 7.44 | 4.4 | <.0001 | 80.2 |
|  | rep | 1 | 31.76 | 31.76 | 18.8 | <.0001 |  |
|  | Error | 208 | 351.01 | 1.69 |  |  |  |
| AlSFW | Genotype | 208 | 8449339.14 | 40621.82 | 41.1 | <.0001 | 96.9 |
|  | rep | 1 | 63360.93 | 63360.93 | 64.1 | <.0001 |  |
|  | Error | 208 | 205663.36 | 988.77 |  |  |  |
| AlSDW | Genotype | 208 | 127445.86 | 612.72 | 25.5 | <.0001 | 94.9 |
|  | rep | 1 | 1864.13 | 1864.13 | 77.5 | <.0001 |  |
|  | Error | 208 | 5005.78 | 24.07 |  |  |  |
| AlRDW | Genotype | 208 | 5967.46 | 28.69 | 14.2 | <.0001 | 92.8 |
|  | rep | 1 | 42.43 | 42.43 | 21.0 | <.0001 |  |
|  | Error | 208 | 419.64 | 2.02 |  |  |  |
| AlSWC | Genotype | 208 | 267871.70 | 1287.84 | 2426.0 | <.0001 | 99.9 |
|  | rep | 1 | 7.92 | 7.92 | 14.9 | 0.0001 |  |
|  | Error | 208 | 110.42 | 0.53 |  |  |  |
| Al/CKSH | Genotype | 208 | 4.89 | 0.02 | 9.4 | <.0001 | 90.3 |
|  | rep | 1 | 0.00 | 0.00 | 0.3 | 0.5633 |  |
|  | Error | 208 | 0.52 | 0.00 |  |  |  |
| Al/CKRL | Genotype | 208 | 18.21 | 0.09 | 2.9 | <.0001 | 72.9 |
|  | rep | 1 | 0.43 | 0.43 | 14.0 | 0.0002 |  |
|  | Error | 208 | 6.35 | 0.03 |  |  |  |
| Al/CKSFW | Genotype | 208 | 9.46 | 0.05 | 6.1 | <.0001 | 85.2 |
|  | rep | 1 | 0.08 | 0.08 | 11.3 | 0.0009 |  |
|  | Error | 208 | 1.56 | 0.01 |  |  |  |
| Al/CKSDW | Genotype | 208 | 12.87 | 0.06 | 4.8 | <.0001 | 78.9 |
|  | rep | 1 | 0.74 | 0.74 | 56.7 | <.000 |  |
|  | Error | 208 | 2.71 | 0.01 |  |  |  |
| Al/CKRDW | Genotype | 208 | 70.80 | 0.34 | 9.1 | <.0001 | 88.3 |
|  | rep | 1 | 1.54 | 1.54 | 41.1 | <.0001 |  |
|  | Error | 208 | 7.82 | 0.04 |  |  |  |
| Al/CKSWC | Genotype | 208 | 0.35 | 0.00 | 14.5 | <.0001 | 92.6 |
|  | rep | 1 | 0.00 | 0.00 | 31.1 | <.0001 |  |
|  | Error | 208 | 0.02 | 0.00 |  |  |  |
