# Supplementary material for: Genome-wide association mapping of aluminum toxicity tolerance and fine mapping of a candidate gene for Nrat1 in rice
Source: PLoS One. 2018 Jun 12;13(6):e0198589. doi: 10.1371/journal.pone.0198589 (PMC5997306; doi:10.1371/journal.pone.0198589)
Supplement: S3 Table — (DOCX) [file pone.0198589.s007.docx]

| **Sample** | **Raw**  **base (bp)** | **Clean**  **base (bp)** | **Effective** | **Q20 (%)** | **Mapped**  **reads** | **Total**  **reads** | **Mapping** | **Average** |
| --- | --- | --- | --- | --- | --- | --- | --- | --- |
|  |  |  | **rate (%)** |  |  |  | **rate (%)** | **Depth (X)** |
| PR | 5,174,010,600 | 5,170,698,000 | 99.94 | 95.73 | 32,889,856 | 34,471,320 | 95.4 | 13 |
| PS | 5,151,057,000 | 5,147,333,700 | 99.93 | 95.88 | 33,233,556 | 34,315,558 | 96.9 | 13.1 |
| BR | 9,322,114,500 | 9,315,804,600 | 99.93 | 95.84 | 59,695,161 | 62,105,364 | 96.1 | 22.6 |
| BS | 9,038,503,500 | 9,032,635,800 | 99.94 | 96.06 | 57,849,042 | 60,217,572 | 96.1 | 21.8 |

**S3 Table.** Statistic of sequencing results
