# Supplementary material for: Genome-wide association mapping of aluminum toxicity tolerance and fine mapping of a candidate gene for Nrat1 in rice
Source: PLoS One. 2018 Jun 12;13(6):e0198589. doi: 10.1371/journal.pone.0198589 (PMC5997306; doi:10.1371/journal.pone.0198589)
Supplement: S4 Table — (DOCX) [file pone.0198589.s008.docx]

**S4 Table. List of 6 SNP with △SNP index equal to 1 and neared genes**

| **SNP type** | **POS** | **Ref** | **PR** | **PS** | **Neared gene** | **Gene annotation** |
| --- | --- | --- | --- | --- | --- | --- |
| upstream | 938008 | C | G | C | *LOC_Os02g02580* | PC-Esterase\|\|PMR5_N_dom\|\|TBL\|\|TBL12 |
| downstream | 938008 | C | G | C | *LOC_Os02g02590* | PPR repeat domain containing protein |
| upstream | 1375515 | A | A | G | *LOC_Os02g03390* | expressed protein |
| upstream | 1375533 | A | A | G | *LOC_Os02g03390* | expressed protein |
| upstream | 1433066 | A | A | G | *LOC_Os02g03530* | Pentatricopeptide_repeat\|\|TPR-like_helical |
| upstream | 2367161 | C | C | T | *LOC_Os02g04990* | expressed protein |
| Intergenic | 2372708 | C | T | C | *LOC_Os02g05000* | expressed protein |
| Intergenic | 2372708 | C | T | C | *LOC_Os02g05020* | hypothetical protein |
